# Technology-enhanced practice competencies: scoping review and novel model development

**DOI:** 10.3389/fdgth.2025.1571518

**Published:** 2025-04-25

**Authors:** Jonathan G. Perle, Dilip N. Chandran, Emily Brezler, Michelle Coleman, Julia Deziel, Patrick Nahhas, Gabrielle McDonald, Jason F. Jent

**Affiliations:** ^1^ Department of Behavioral Medicine and Psychiatry, Rockefeller Neuroscience Institute, West Virginia University School of Medicine, Morgantown, WV, United States; ^2^ Department of Internal Medicine and Pediatrics, University of Louisville School of Medicine, Louisville, KY, United States; ^3^ West Virginia University School of Medicine, Morgantown, WV, United States; ^4^ Department of Behavioral Medicine and Psychiatry, West Virginia University School of Medicine, Morgantown, WV, United States; ^5^ Department of Pediatrics, Leonard M. Miller School of Medicine, University of Miami, Miami, FL, United States

**Keywords:** telehealth, telemedicine, technology, competency, training, education

## Abstract

**Introduction:**

With technology routinely integrated into healthcare, it is essential that practitioners obtain skills in the numerous competencies required. Unfortunately, literature to guide use remains inconsistent and fragmented. The current scoping review identified technology-enhanced practice competencies for healthcare practitioners among peer-reviewed literature.

**Methods:**

A review of PubMed, Scopus, Web of Science, PsycInfo, Global Index Medicus, and Journal of Technology in Behavioral Science was conducted between November 2022 and March 2023.

**Results:**

10,583,799 articles were identified, with 109 included in the final review. Seventeen primary competencies were identified with ethics (77.1%), legality (68.8%), and data security (65.1%) among the top three.

**Conclusions:**

Although multiple technologies across specialties were identified, limited literature comprehensively defined technology-enhanced practice competencies to guide practitioner education. To address this gap, the Intersectional Technology Education and Competency in Healthcare (iTECH) Model was created to clarify educational targets for the use of technology in healthcare practices. Model development and finding applications are discussed.

## Introduction

Technology-enhanced practices, broadly defined for the current study as practices involving practitioner and patient interactions with a technology that includes some level of practitioner involvement and/or oversight for the purpose of healthcare-related information collection or intervention services (1), have been utilized in healthcare for over a century (2). Such practices are categorized as synchronous (i.e., live, interactive), asynchronous (i.e., non-live), or hybrid (i.e., combination of synchronous, asynchronous, and in-person) (3, 4). Despite use, adoption among healthcare specialties (e.g., medicine, psychology, nursing, social work, counseling, physical therapy, occupational therapy) was suggested as relatively slow (5). While universally-accepted reasons for slow adoption are not well-defined, hypothesized reasons include limited training in the technologies leading to a lack of comfort, financial barriers to implementation, and a lack of organizational infrastructure to support the ongoing use of technologies (6–9). Limited usage continued until the late 1990s and early 2000s; coinciding with technology becoming smaller, cheaper, more powerful, more readily accessible, and more interconnected (10, 11). Among all technologies, telecommunication technologies uniquely demonstrated an unexpected and unprecedented growth in integration and expansion in response to the COVID-19 pandemic (12, 13). Expansion across time, combined with both practitioner and patient satisfaction (14–16) suggested that the integration of technologies into healthcare services is not only here to stay, but warrants clarification of relevant competencies to ensure that healthcare practitioners are effectively harnessing the technologies within their practices.

A cursory review of the technology-focused competency literature suggests the large emphasis on telehealth, or the integration of telecommunication technologies with healthcare services (e.g., videoconferencing, telephone, email, messaging programs), which has frequently been heralded as the future of medical and mental health-related healthcare (17, 18). While still considered limited, telehealth literature across healthcare specialties has demonstrated attempts to standardize competencies, including consolidated discussion by the American Telemedicine Association (19–21), the American Psychological Association (22), the American Psychiatric Association (23), and the American Medical Association (24). Nevertheless, literature remains fragmented, as well as varying in focus and elaboration by resource. More specifically, competencies across discussions have included, but are not limited to: awareness of research related to technologies, methods of adapting in-person strategies for digital administration, ethics of practice, legality, data security, troubleshooting technology, interpersonal skills, and interprofessional communication (11, 25–28).

While a positive first step, the landscape of healthcare technology has rapidly evolved beyond the narrow confines of telehealth alone. Recent literature underscores the research-validated utility of a diverse array of technologies in patient care, including virtual/augmented/extended reality (VR, AR, XR) (29, 30); robotics (31), video games (32), wearable technologies (33), artificial intelligence (AI) (34), and web-based self-guided assessment and intervention packages (35). This expansion necessitates a broader conceptualization of technology-enhanced practices that extends far beyond telecommunication alone.

Unfortunately, the rapid evolution of healthcare technology outpaced current educational paradigms, creating a critical gap between innovation and practitioner competencies. This disparity threatens the ethical, legal, evidence-informed, and safe integration of novel technologies into patient care (11). Proficient use of technology in healthcare demands more than both general knowledge and applied skills; it requires a nuanced understanding of diverse applications across various settings and populations. Simply put, being an excellent practitioner and adept at general technology use does not necessarily make one readily able to successfully integrate novel technologies into healthcare practices due to the large number of unique and unknown challenges that may arise.

As guiding healthcare organizations, ethical codes, and regulatory/licensing boards continue to promote evidence-informed education for technology-enhanced practice, clarification of relevant competencies to guide integration and continuing education remains prudent. Towards this end, there remains an urgent need for evaluation of available evidence-informed recommendations that address the broad spectrum of technology-enhanced practices in healthcare to inform practitioners' judicious use of these diverse technologies and ensure their effective integration into clinical practices. This endeavor can identify relevant documentation, as well as ongoing field gaps. Unfortunately, to date, no known work has evaluated the literature for technology-enhanced practice competencies (beyond telehealth), either independently or across healthcare specialties. This study aims to address this notable gap in the literature by conducting a scoping review of technology-enhanced practice competencies among peer-reviewed literature across healthcare specialties. Utilizing a comprehensive approach, we examine synchronous, asynchronous, and hybrid practitioner-patient interactions within various technological contexts. The investigation is guided by two primary research questions: (1) Which technology types are discussed in competency frameworks across healthcare specialties, and (2) What technology-enhanced practice competencies are recommended in the literature to guide practitioner use? By synthesizing findings from peer-reviewed sources, this study seeks to provide insights into the current landscape of technology competencies in healthcare, and may inform the development of more cohesive, multiprofessional approaches to technology integration in clinical practice.

## Methods

### Identifying relevant studies and study selection

The review utilized the Preferred Reporting Items for Systematic Review and Meta-Analysis extension for Scoping Reviews (PRISMA-ScR) reporting standards (36) (Supplementary Figure S1), as well as published scoping review methodologies (37, 38). More specifically, as based upon the study's primary questions, a modified population, concept, and context (PCC) framework was utilized in which the population was defined more broadly as healthcare specialties rather than specific population characteristics (e.g., age, race), concept was defined broadly as technology competencies, and the context included the setting of the technology use (39, 40). PubMed, Scopus, Web of Science, PsycInfo, and Global Index Medicus were reviewed between November 2022 and March 2023 (see Supplementary Table S1 for Boolean operators). Due to a high number of competency- and training-focused works being published in the Journal of Technology in Behavioral Science, yet not all works being identified on searched databases, this journal was also specifically reviewed with the same search methodology. Rayyan, a web-based application for conducting structured literature reviews, was utilized to organize data and remove duplicates (41).

#### Inclusionary criteria

Following the removal of duplicates, an item was included in the final dataset if it was written in English, was a manuscript in a peer-reviewed journal, focused on technology, focused on healthcare, focused on the education of a healthcare practitioner (i.e., graduate-level training through licensed professional), and included a direct naming of a specific competency or educational target combined with at least one statement defining/detailing the competency (i.e., a manuscript stating “data security” was not included, while a manuscript saying “data security” and also detailing that this is inclusive of passwords and/or encryption standards was included). This approach was designed to eliminate papers that were merely listing topics, and thus less helpful for practitioners seeking applied knowledge for their practices. A competency was defined for the current review as a designated target of practitioner knowledge and/or applied skill for the specific technology to ensure an ethical, legal, safe, and evidence-informed service. This definition aligns with similar review literature defining the term competency or competencies (33, 42–46). As the use of technology in healthcare can be traced back to the 1800s (2), to ensure comprehensive review, no year-related criteria were applied (i.e., all manuscripts through March 2023 were eligible for inclusion).

#### Data cleaning and screening processes

In line with suggestions for screening very large amounts of data, a title-first approach was utilized (47). This approach has been suggested as more efficient, yet comparable to screening both titles and abstracts together. To account for Rayyan's lack of sequential Boolean operator-based screening, titles were first screened by education-, teaching-, and training-related keywords; then technology-related keywords; and finally, healthcare-related keywords for relevancy. Standardized keywords were collectively identified by the authors as relevant to the scoping review (Supplementary Table S2). Among the remaining items, abstracts were screened for additional applicability. Each potential item was reviewed by three sets of two authors, with a third author as a tie breaker, as needed. Following training by the primary author, interrater reliability kappa values for all pairs were ≥0.99, suggesting “almost perfect” levels of agreement (48). Finally, full texts of remaining items were screened and coded to identify technology-focused competencies (e.g., ethics, legality) relevant to healthcare services. Coding was completed by the two first authors to establish consensus. Prior to discussion of disagreements until consensus was reached (42), interrater reliability kappa value was 0.93 for overall agreement for inclusion/exclusion of each identified manuscript.

As further detailed in Table 1, manuscripts were coded across the variables of: paper type, publication date, author location, specialty area, whether the discussion was interdisciplinary (i.e., discussed more than one specialty), career stage, location of discussion, types of technology, and identified competencies.

**Table 1:** Frequencies of coded variables Among included manuscripts (*N* = 109).

Year published	Number of manuscripts	Percentage of total (*N =* 109)
2000	1	0.9%
2002	1	0.9%
2003	1	0.9%
2004	1	0.9%
2005	4	3.7%
2006	1	0.9%
2008	3	2.8%
2010	1	0.9%
2011	6	5.5%
2012	5	4.6%
2013	4	3.7%
2014	4	3.7%
2015	7	6.4%
2016	3	2.8%
2017	5	4.6%
2018	5	4.6%
2019	5	4.6%
2020	13	11.9%
2021	25	22.9%
2022	11	10.1%
2023	3	2.8%
Author location^a^^,^^b^	Number of manuscripts	Percentage of total (*N =* 109)
Algeria	1	0.9%^d^
Australia	9	8.3%^d^
Bahrain	1	0.9%^d^
Canada	8	7.3%^d^
Egypt	1	0.9%^d^
India	1	0.9%^d^
Iran	1	0.9%^d^
Iraq	1	0.9%^d^
Italy	1	0.9%^d^
Jordan	1	0.9%^d^
Kuwait	1	0.9%^d^
Lebanon	1	0.9%^d^
Libya	1	0.9%^d^
Morocco	1	0.9%^d^
Northern Ireland	1	0.9%^d^
Palestine	1	0.9%^d^
Qatar	1	0.9%^d^
Saudi Arabia	3	2.8%^d^
South Africa	3	2.8%^d^
Spain	1	0.9%^d^
Syria	1	0.9%^d^
Switzerland	2	1.8%^d^
The Netherlands	1	0.9%^d^
Tunisia	1	0.9%^d^
United Arab Emirates	1	0.9%^d^
United Kingdom	2	1.8%^d^
United States	86	78.9%^d^
Type of paper	Number of manuscripts	Percentage of total (*N =* 109)
Quantitative	14	12.8%
Qualitative	91	83.5%
Mixed Method	4	3.7%
Type of quantitative paper^e^	Number of manuscripts	Percentage of sub-total (*N =* 14)
One-Time Survey or Assessment	6	42.9%
Pre-Post Test or Multiple Time Points Survey or Assessment	8	57.1%

Type of qualitative paper^e^	Number of manuscripts	Percentage of sub-total (*N =* 91)
General Description, Viewpoint, Discussion, Qualitative Analysis, or Non-Structured Review	71	78.0%
Program-Specific Outline or Discussion	10	11.0%
Review Article	10	11.0%
Type of mixed method paper^e^	Number of manuscripts	Percentage of sub-total (*N =* 4)
One-Time Survey or Assessment and Qualitative Output	1	25.0%
Pre-Post Test or Multiple Time Points Survey or Assessment, and Qualitative Output	3	75.0%
Specialties^a^^,^^c^	Number of manuscripts	Percentage of total (*N =* 109)
Addition Medicine	4	3.7%^d^
Allergy	1	0.9%^d^
Assistive Technology	1	0.9%^d^
Audiology	1	0.9%^d^
Behavior Analysis	7	6.4%^d^
Cardiology	1	0.9%^d^
Counseling	13	11.9%^d^
Dermatology	1	0.9%^d^
Dietic/Nutrition	2	1.8%^d^
Emergency Medicine	1	0.9%^d^
Family Medicine	1	0.9%^d^
Genetics	1	0.9%^d^
Immunology	1	0.9%^d^
Marriage and Family Therapy	12	11.0%^d^
Music Therapy	1	0.9%^d^
Nephrology	1	0.9%^d^
Neurology	6	5.5%^d^
Neuropsychology	1	0.9%^d^
Nursing	14	12.8%^d^
Nurse Practitioner	7	6.4%^d^
Occupational Therapy	4	3.7%^d^
Oncology	2	1.8%^d^
Orthopedic	1	0.9%^d^
Orthotist	1	0.9%^d^
Pathology	1	0.9%^d^
Pediatrics	3	2.8%^d^
Pharmacy	3	2.8%^d^
Physical Therapy	4	3.7%^d^
Psychiatry	28	25.7%^d^
Psychology	50	45.9%^d^
Radiology	1	0.9%^d^
Rehabilitation Medicine	1	0.9%^d^
Rheumatology	4	3.7%^d^
Social Work	21	19.3%^d^
Speech Therapy	4	3.7%^d^
Surgery	2	1.8%^d^
Urology	1	0.9%^d^
Interdisciplinary discussion^c^	Number of manuscripts	Percentage of total (*N =* 109)
Yes	22	20.2%
Career stage focus^a^^,^^c^	Number of manuscripts	Percentage of total (*N =* 109)
Graduate Level Trainee/Student	34	31.2%^d^
Licensed Practitioner	75	68.8%^d^
Non-Professional Health Operator	1	0.9%^d^
Paraprofessional	1	0.9%^d^
Resident, Intern, Fellow	28	25.7%^d^

Location of discussion^a^^,^^c^	Number of manuscripts	Percentage of total (*N =* 109)
College Counseling	1	0.9%^d^
Home-Based	1	0.9%^d^
Medical Center	6	5.5%^d^
Primary Care	2	1.8%^d^
School/Academic	15	13.8%^d^
University Outpatient Clinic	1	0.9%^d^
Veterans Affairs	1	0.9%^d^
Type of technology discussed^a^^,^^c^	Number of manuscripts	Percentage of total (*N =* 109)
App	29	26.6%^d^
Artificial Intelligence (AI)	2	1.8%^d^
Email	44	40.4%^d^
Internet (Broadly Defined)	1	0.9%^d^
Messaging Program	35	32.1%^d^
Nanomachine	1	0.9%^d^
Robotic	2	1.8%^d^
Social Media	19	17.4%^d^
Telephone	62	56.9%^d^
Video	91	83.5%^d^
Video Game	1	0.9%^d^
Wearable	11	10.1%^d^
Web-Based Assessment or Intervention	12	11.0%^d^
VR, AR, XR	3	2.8%^d^
Competencies discussed^a^^,^^c^	Number of manuscripts	Percentage of total (*N =* 109)
Adaptations of Assessment	44	40.4%^d^
Adaptations of Communication	52	47.7%^d^
Adaptations of Intervention	25	22.9%^d^
Administrative Responsibilities	42	38.5%^d^
Data Security	71	65.1%^d^
Diversity, Equity, Inclusion	32	29.4%^d^
Ethics	84	77.1%^d^
Interprofessional Collaboration	15	13.8%^d^
Legal	75	68.8%^d^
Appropriateness Evaluation	41	37.6%^d^
Patient Safety	42	38.5%^d^
Professionalism	42	38.5%^d^
Learning/Teaching Others	23	21.1%^d^
Research	1	0.9%^d^
Self-Care	1	0.9%^d^
Techno-Etiquette	60	55.0%^d^
Troubleshooting	27	24.8%^d^

^a^ Some manuscripts had multiple applicable selections.

^b^ Author affiliation as provided on manuscript.

^c^ Not all papers clearly defined characteristics. Only those with clear indications of relevant variables were included in this coding scheme.

^d^ Given that manuscripts could have multiple selections per variable, numbers are relative to the total and may not add to 100%.

^e^ “One-time Survey” was defined as a survey completed at one time-point only; “Pre-Post Test or Survey” was defined as an assessment or survey completed before and after an implemented process (e.g., intervention); “General Description, Viewpoint, Discussion, Qualitative Analysis, or Non-Structured Review” was defined as a description, viewpoint, discussion, analysis, or review of a field or specific topic without a formal review methodology, detailed description of a program, or quantitative analysis; “Program-Specific Outline or Discussion” was defined as a description, outline, or general discussion of a specific program, or program implementation, without quantitative analysis of the program; “Review Article” was defined as an article reviewing a field or specific topic in detail through the defining of database and search criteria, including systematic, scoping, or narrative review methodologies.

## Results

The initial search yielded 10,583,799 records (Supplementary Figure S1). One hundred and nine met inclusionary criteria and were included in the final review (Tables 1, 2).

**Table 2:** Characteristics of included studies (*N* = 109)^a^.

Author, affiliation, and year	Type of paper	Specialty area	Career stage focus	Setting	Technology type	Identified competencies^b^
• Abbott et al. • Australia • 2008	• Qualitative: General Description, Viewpoint, Discussion, Qualitative Analysis, or Non-Structured Review	• Psychology	• Licensed Practitioner	• None	• E-Mail • Messaging Program • Telephone	• AC, AE, AI, DS, E, L, P, T-E
• Alkureishi et al. • United States • 2021	• Qualitative: General Description, Viewpoint, Discussion, Qualitative Analysis, or Non-Structured Review	• None	• Graduate Level Trainee/Student • Licensed Practitioner • Resident, Intern, Fellow	• None	• Telephone • Video	• AC, T-E
• Almubark et al. • Saudi Arabia • 2022	• Qualitative: General Description, Viewpoint, Discussion, Qualitative Analysis, or Non-Structured Review	• Occupational Therapy • Physical Therapy • Speech Therapy	• Licensed Practitioner	• None	• E-Mail • Telephone • Video	• AA, AE, E
• Arends et al. • United States • 2021	• Quantitative: Pre-Post Test or Multiple Time Points Survey or Assessment	• Nurse Practitioner	• Graduate Level Trainee/Student	• School/Academic	• None	• AA, AC, AR, E, L, P, T-E
• Armstrong • United States • 2019	• Quantitative: Pre-Post Test or Multiple Time Points Survey or Assessment	• Counseling • Nursing • Pharmacy • Psychology • Social Work	• Licensed Practitioner	• None	• App • Telephone	• AC, AI, AR, DEI, DS, E
• Baker and Bufka • United States • 2011	• Qualitative: General Description, Viewpoint, Discussion, Qualitative Analysis, or Non-Structured Review	• Psychology	• Licensed Practitioner	• None	• E-Mail • Video	• AR, DS, E, L
• Baltimore • United States • 2000	• Qualitative: General Description, Viewpoint, Discussion, Qualitative Analysis, or Non-Structured Review	• Counseling	• Licensed Practitioner	• None	• E-Mail • Video	• DS, E
• Baumes et al. • United States • 2020	• Qualitative: General Description, Viewpoint, Discussion, Qualitative Analysis, or Non-Structured Review	• Behavior Analysis • Pediatrics • Psychology • Social Work	• Licensed Practitioner	• None	• None	• AE, AI, AR, DEI, DS, E, L, PS, T-E
• Brimley et al. • United States • 2021	• Qualitative: Review Article	• Urology	• Licensed Practitioner	• None	• App • Telephone • Video	• AE, AR, DS, E, L, T-E
• Casline et al. • United States • 2021	• Qualitative: General Description, Viewpoint, Discussion, Qualitative Analysis, or Non-Structured Review	• Psychology	• Graduate Level Trainee/Student	• School/Academic	• Video	• AA, E, L/T, T-E
• Caver et al. • United States • 2020	• Qualitative: Program- Specific Outline or Discussion	• Psychiatry • Psychology • Social Work	• Licensed Practitioner	• Veterans Affairs	• Video	• E, L/T, L, PS, T
• Chike-Harris et al. • United States • 2022	• Mixed Method: Pre-Post Test or Multiple Time Points Survey or Assessment, and Qualitative Output	• Nursing • Nurse Practitioner	• Graduate Level Trainee/Student • Licensed Practitioner • Resident, Intern, Fellow	• School/Academic	• Video	• AC, DS, P, T-E
• Chipps et al. • South Africa • 2012	• Qualitative: General Description, Viewpoint, Discussion, Qualitative Analysis, or Non-Structured Review	• Psychiatry	• Licensed Practitioner	• None	• Video	• AE, AR, DS, L, T-E
• Cooper et al. • United States • 2019	• Qualitative: General Description, Viewpoint, Discussion, Qualitative Analysis, or Non-Structured Review	• Psychology	• Licensed Practitioner	• None	• E-Mail • Telephone • Video	• AC, AE, AI, AR, DEI, DS, E, L/T, L, P, T-E, T
• Costich et al. • United States • 2021	• Quantitative: Pre-Post Test or Multiple Time Points Survey or Assessment	• Pediatrics	• Resident, Intern, Fellow	• Medical Center	• Video	• T-E
• Daniel and Sulmasy • United States • 2015	• Qualitative: General Description, Viewpoint, Discussion, Qualitative Analysis, or Non-Structured Review	• None	• Licensed Practitioner • Resident, Intern, Fellow	• Primary Care	• None	• AE, DEI, DS, E, L
• de Leo et al. • Italy • Switzerland • 2003	• Qualitative: Program- Specific Outline or Discussion	• Emergency Medicine	• Non-Professional Health Operator	• Medical Center	• VR, AR, XR	• AI, T-E
• DeJong • United States • 2014	• Qualitative: General Description, Viewpoint, Discussion, Qualitative Analysis, or Non-Structured Review	• Psychiatry	• Licensed Practitioner	• None	• Social Media • Telephone	• AR, DS, E, L, P, PS, T-E
• DeJong et al. • United States • 2012	• Qualitative: General Description, Viewpoint, Discussion, Qualitative Analysis, or Non-Structured Review	• Psychiatry	• Resident, Intern, Fellow	• None	• E-Mail • Social Media	• AC, DS, E, L, P
• DeJong et al. • United States • 2015	• Qualitative: General Description, Viewpoint, Discussion, Qualitative Analysis, or Non-Structured Review	• None	• None	• None	• None	• AA, AC, AE, IC, L
• Dopp et al. • United States • 2021	• Mixed Method: One-Time Survey or Assessment and Qualitative Output	• Psychology	• Graduate Level Trainee/Student	• School/Academic	• App • E-Mail • Messaging Program • Telephone • Video • Web-Based Assessment or Intervention	• AC
• Drude et al. • United States • 2020	• Qualitative: General Description, Viewpoint, Discussion, Qualitative Analysis, or Non-Structured Review	• Addiction Medicine • Behavior Analysis • Counseling • Marriage/Family Therapy • Nursing • Nurse Practitioner • Psychiatry • Psychology • Social Work	• Graduate Level Trainee/Student	• None	• App • Social Media • Telephone • Video	• AC, AR, IC, E, L/T, L, T-E
• Drum & Littleton • United States • 2014	• Qualitative: General Description, Viewpoint, Discussion, Qualitative Analysis, or Non-Structured Review	• Psychology	• Licensed Practitioner	• None	• App • E-Mail • Messaging Program • Telephone • Video • Web-Based Assessment or Intervention	• AC, AR, E, L, P, T-E
• Farmer et al. • United States • 2020	• Qualitative: General Description, Viewpoint, Discussion, Qualitative Analysis, or Non-Structured Review	• Psychology	• Licensed Practitioner	• None	• Video	• AA, AC, AE, DEI, E, L, T-E
• Fitzgerald et al. • Canada • United States • 2010	• Qualitative: General Description, Viewpoint, Discussion, Qualitative Analysis, or Non-Structured Review	• Psychology	• None	• None	• E-Mail • Messaging Program • Telephone • Video	• DS, E, L, P, PS
• Frankl et al. • United States • 2021	• Mixed Method: Pre-Post Test or Multiple Time Points Survey or Assessment, and Qualitative Output	• None	• Graduate Level Trainee/Student	• School/Academic	• Video	• AA, AE, AI, DEI, P, T-E, T
• Fuertes-Guiró and Velasco • Spain • 2018	• Qualitative: General Description, Viewpoint, Discussion, Qualitative Analysis, or Non-Structured Review	• Surgery	• Licensed Practitioner	• None	• Robotic • Video	• AI, DS, E, L/T, L, T-E
• Gibson et al. • United States • 2021	• Qualitative: General Description, Viewpoint, Discussion, Qualitative Analysis, or Non-Structured Review	• Nursing	• Graduate Level Trainee/Student	• School/Academic	• Video	• AC, T-E, T
• Gifford et al. • United States • 2012	• Quantitative: Pre-Post Test or Multiple Time Points Survey or Assessment	• Counseling • Psychology • Social Work	• Licensed Practitioner • Paraprofessional	• None	• Telephone • Video	• E, L, PS, T-E
• Govindarajan et al. • United States • 2017	• Qualitative: General Description, Viewpoint, Discussion, Qualitative Analysis, or Non-Structured Review	• Neurology	• Graduate Level Trainee/Student • Licensed Practitioner • Resident, Intern, Fellow	• None	• Video	• AA, AR, DS, E, L/T, L, P, T-E
• Hames et al. • Canada • United States • 2020	• Quantitative: One-Time Survey or Assessment	• Psychology	• Graduate Level Trainee/Student	• None	• Telephone • Video	• AA, AC, AE, AI, AR, DEI, DS, E, L/T, L, SC, T-E, T
• Hart et al. • United States • 2022	• Quantitative: Pre-Post Test or Multiple Time Points Survey or Assessment	• None	• Graduate Level Trainee/Student	• None	• Telephone • Video	• AA, AC, AE, AR, DEI, E, IC, L, P, PS, T
• Haydon et al. • Australia • 2021	• Qualitative: General Description, Viewpoint, Discussion, Qualitative Analysis, or Non-Structured Review	• Psychology	• Licensed Practitioner	• None	• E-Mail • Messaging Program • Video	• AC, AE, DS, E, P, PS, T-E, T
• Hertlein et al. • United States • 2021	• Qualitative: General Description, Viewpoint, Discussion, Qualitative Analysis, or Non-Structured Review	• Marriage/Family Therapy	• Graduate Level Trainee/Student • Licensed Practitioner	• None	• App • E-Mail • Messaging Program • Social Media • Telephone • Video	• AA, AC, AE, AI, AR, E, L, P, PS, T-E, T
• Hertlein et al. • United States • 2021	• Qualitative: General Description, Viewpoint, Discussion, Qualitative Analysis, or Non-Structured Review	• Marriage/Family Therapy	• Licensed Practitioner	• None	• App • Artificial Intelligence • Social Media • Telephone • Video • Wearable	• AC, AE, E, L/T, L, T-E
• Hilty et al. • United States • 2019	• Qualitative: General Description, Viewpoint, Discussion, Qualitative Analysis, or Non-Structured Review	• Psychiatry	• Licensed Practitioner • Resident, Intern, Fellow	• None	• App • E-Mail • Messaging Program • Telephone • Video	• AA, AC, AE, AI, AR, DEI, DS, E, L/T, L, P, PS, T-E, T
• Hilty et al. • United States • 2020	• Qualitative: Review Article	• Behavior Analysis • Counseling • Marriage/Family Therapy • Nursing • Psychiatry • Psychology • Social Work	• None	• None	• App • E-Mail • Messaging Program • Social Media • Telephone • Video • Wearable	• AA, AC, E, L/T, P, PS, T-E, T-E
• Hilty et al. • Canada • United States • 2015	• Qualitative: General Description, Viewpoint, Discussion, Qualitative Analysis, or Non-Structured Review	• Psychiatry	• Graduate Level Trainee/Student • Licensed Practitioner • Resident, Intern, Fellow	• None	• Video	• AA, AC, AI, AR, DEI, DS, E, L, P, PS, T-E, T
• Hilty et al. • United States • 2017	• Qualitative: General Description, Viewpoint, Discussion, Qualitative Analysis, or Non-Structured Review	• Behavior Analysis • Counseling • Marriage and Family Therapy • Psychiatry • Psychology • Social Work	• Graduate Level Trainee/Student • Licensed Practitioner • Resident, Intern, Fellow	• None	• App • E-Mail • Messaging Program • Telephone • Video	• AA, AC, AR, DEI, DS, E, IC, L, P, PS
• Hilty et al. • Canada • United States • 2018	• Qualitative: General Description, Viewpoint, Discussion, Qualitative Analysis, or Non-Structured Review	• Psychiatry	• Resident, Intern, Fellow	• None	• App • E-Mail • Messaging Program • Social Media • Telephone • Video • Wearable • Web-Based Assessment or Intervention	• AA, AC, AR, DEI, DS, E, IC, L/T, L, P, PS, T-E
• Hilty et al. • United States • 2021	• Qualitative: Review Article	• Psychiatry	• Licensed Practitioner • Resident, Intern, Fellow	• None	• App • E-Mail • Messaging Program • Telephone • Video • Wearable	• AA, AC, AE, AI, AR, DS, E, IC, L/T, L, P, PS, T-E, T
• Hilty et al. • United States • 2021	• Qualitative: Review Article	• Counseling • Marriage/Family Therapy • Psychiatry • Psychology • Social Work	• Graduate Level Trainee/Student • Licensed Practitioner • Resident, Intern, Fellow	• None	• App • E-Mail • Messaging Program • Social Media • Telephone • Video • Wearable • Web-Based Assessment or Intervention	• AA, AC, AE, AI, AR, DEI, DS, E, L/T, L, P, PS, T
• Jagolino et al. • United States • 2016	• Quantitative: One-Time Survey or Assessment	• Neurology	• Licensed Practitioner	• None	• None	• AA, AC, AI, IC, P, T
• Jarvis-Selinger et al. • Canada • 2008	• Qualitative: Review Article	• Cardiology • Dermatology • Family Medicine • Genetics • Nephrology • Neurology • Nursing • Occupational Therapy • Orthopedics • Pathology • Pediatrics • Pharmacy • Physical Therapy • Psychiatry • Radiology • Rehabilitation • Rheumatology • Social Work • Speech Therapy • Surgery	• Licensed Practitioner	• None	• Video	• DS, IC, L, T-E, T
• Johnson^7^ • Canada • 2014	• Qualitative: General Description, Viewpoint, Discussion, Qualitative Analysis, or Non-Structured Review	• Psychology	• Licensed Practitioner	• None	• App • E-Mail • Messaging Program • Telephone • Video	• AA, AC, AE, AI, DS, E, L, P, PS, T-E
• Joint Task Force for the Development of Telepsychology Guidelines for Psychologists • United States • 2013	• Qualitative: General Description, Viewpoint, Discussion, Qualitative Analysis, or Non-Structured Review	• Psychology	• Licensed Practitioner	• None	• E-Mail • Messaging Program • Social Media • Telephone • Video • Web-Based Assessment or Intervention	• AA, AE, AR, DS, E, L, P, PS
• Jones et al. • United Kingdom • 2006	• Qualitative: General Description, Viewpoint, Discussion, Qualitative Analysis, or Non-Structured Review	• Psychiatry	• Licensed Practitioner	• None	• Video	• AC, DS, E, T-E, T
• Karcher and Presser • United States • 2016	• Qualitative: General Description, Viewpoint, Discussion, Qualitative Analysis, or Non-Structured Review	• Psychology	• Licensed Practitioner	• None	• App • Messaging Program • Telephone • Video	• AA, AE, AR, DEI, DS, E, L/T, L, P, PS
• Keswani et al. • United States • 2020	• Qualitative: General Description, Viewpoint, Discussion, Qualitative Analysis, or Non-Structured Review	• Allergy • Immunology	• Graduate Level Trainee/Student • Resident, Intern, Fellow	• School/Academic	• Messaging Program • Telephone • Video	• AA, AC, DS, L, P, T-E
• Khan et al. • United States • 2021	• Quantitative: One-Time Survey or Assessment	• Psychiatry	• Graduate Level Trainee/Student • Licensed Practitioner • Resident, Intern, Fellow	• None	• Video	• AA, AC, IC, L, P, PS, T-E
• Khan and Ramtekkar • United States • 2019	• Qualitative: General Description, Viewpoint, Discussion, Qualitative Analysis, or Non-Structured Review	• Psychiatry	• Licensed Practitioner • Resident, Intern, Fellow	• None	• Video	• AC, T-E, T
• Koh et al. • United States • 2013	• Quantitative: One-Time Survey or Assessment	• Psychiatry	• Licensed Practitioner	• None	• E-Mail • Messaging Program • Social Media • Telephone • Video	• E, L, P
• Lockwood et al. • United States • 2022	• Qualitative: General Description, Viewpoint, Discussion, Qualitative Analysis, or Non-Structured Review	• Rheumatology	• None	• None	• Telephone • Video • Wearable	• AA, E, T-E
• Loman et al. • United States • 2021	• Qualitative: Program- Specific Outline or Discussion	• Neuropsychology	• Licensed Practitioner	• Medical Center	• Video	• AA, AE, DS, P, T-E, T
• Lustgarten and Elhai • United States • 2018	• Qualitative: General Description, Viewpoint, Discussion, Qualitative Analysis, or Non-Structured Review	• Psychology	• Licensed Practitioner	• None	• E-Mail • Messaging Program • Telephone • Video	• DS, E, L, P, PS
• Maheu et al. • United States • 2018	• Qualitative: General Description, Viewpoint, Discussion, Qualitative Analysis, or Non-Structured Review	• Addiction Medicine • Behavior Analysis • Counseling • Marriage/Family Therapy • Nursing • Nurse Practitioner • Psychiatry • Psychology • Social Work	• Licensed Practitioner	• None	• Video	• AC, AE, AR, DEI, DS, E, L, P, PS
• Maheu et al. • United States • 2017	• Qualitative: General Description, Viewpoint, Discussion, Qualitative Analysis, or Non-Structured Review	• Addiction Medicine • Behavior Analysis • Counseling • Marriage/Family Therapy • Nursing • Pharmacy • Psychiatry • Psychology • Social Work	• Graduate Level Trainee/Student • Licensed Practitioner • Resident, Intern, Fellow	• None	• App • E-Mail • Messaging Program • Social Media • Telephone • Video • Wearable	• AA, AC, AE, AI, AR, DEI, DS, E, IC, L/T, L, P, PS, T-E, T
• Maheu et al. • United States • 2018	• Qualitative: General Description, Viewpoint, Discussion, Qualitative Analysis, or Non-Structured Review	• Addiction Medicine • Behavior Analysis • Counseling • Marriage/Family Therapy • Nursing • Psychiatry • Psychology • Social Work	• Graduate Level Trainee/Student • Licensed Practitioner • Resident, Intern, Fellow	• None	• App • E-Mail • Messaging Program • Social Media • Telephone • Video • Wearable	• AA, AC, AE, AI, AR, DEI, DS, E, IC, L/T, L, P, PS, T-E, T
• Maheu et al. • United States • 2021	• Qualitative: General Description, Viewpoint, Discussion, Qualitative Analysis, or Non-Structured Review	• Psychology	• Licensed Practitioner	• None	• Telephone • Video	• AA, AC, AI, AR, DEI, DS, E, IC, L/T, L, P, PS, T-E, T
• Mallen et al. • United States • 2005	• Qualitative: General Description, Viewpoint, Discussion, Qualitative Analysis, or Non-Structured Review	• Psychology	• Licensed Practitioner	• None	• E-Mail • Messaging Program • Telephone • Video	• AC, AI, DEI, DS, E, L/T, L, PS
• Martin et al. • United States • 2020	• Qualitative: General Description, Viewpoint, Discussion, Qualitative Analysis, or Non-Structured Review	• Psychology	• Licensed Practitioner	• None	• App • E-Mail • Messaging Program • Social Media • Telephone • Video • Web-Based Assessment or Intervention	• AA, AC, AE, AI, DEI, DS, E, L, P, PS, T-E
• McCord et al. • United States • 2020	• Qualitative: Review Article	• Psychology	• Licensed Practitioner	• None	• App • E-Mail • Messaging Program • Telephone • Video • Web-Based Assessment or Intervention	• AA, AC, AE, AI, AR, DEI, DS, E, IC, L/T, L, P, PS, R, T-E, T
• McCord et al. • United States • 2015	• Qualitative: Program- Specific Outline or Discussion	• Psychology	• Graduate Level Trainee/Student	• University Outpatient Clinic	• Telephone • Video	• AA, AC, AE, AI, DEI, DS, E, IC, L, P, T
• McCrickard and Butler • United States • 2005	• Qualitative: General Description, Viewpoint, Discussion, Qualitative Analysis, or Non-Structured Review	• Counseling	• Licensed Practitioner	• None	• Internet (Broadly)	• AA, DS, E, PS
• McInroy • United States • 2021	• Qualitative: Program- Specific Outline or Discussion	• Social Work	• Graduate Level Trainee/Student	• School/Academic	• App • Messaging Program • Social Media • Telephone	• DS, E, L/T, L, P
• Menzano et al. • United States • 2011	• Qualitative: General Description, Viewpoint, Discussion, Qualitative Analysis, or Non-Structured Review	• None	• Licensed Practitioner	• College Counseling	• Telephone • Video	• AE, AR, DS, E, L, PS, T-E
• Merrill et al. • United States • 2022	• Qualitative: General Description, Viewpoint, Discussion, Qualitative Analysis, or Non-Structured Review	• Social Work	• Licensed Practitioner	• None	• App • E-Mail • Messaging Program • Social Media • Telephone • Video • Wearable	• AR, DS, E, L
• Miller et al. • United States • 2005	• Qualitative: Program- Specific Outline or Discussion	• Psychiatry	• Licensed Practitioner	• None	• E-Mail • Telephone • Video	• DS, E, L, T-E
• Miller et al. • United States • 2008	• Qualitative: Program- Specific Outline or Discussion	• Nursing • Psychiatry • Psychology • Social Work	• Licensed Practitioner	• Medical Center	• Video	• E, L
• Misra et al. • India • 2005	• Qualitative: Review Article	• Neurology	• Licensed Practitioner • Resident, Intern, Fellow	• School/Academic	• Telephone • Video	• DS
• Murphy and Pomerantz • United States • 2016	• Qualitative: General Description, Viewpoint, Discussion, Qualitative Analysis, or Non-Structured Review	• Psychology	• Licensed Practitioner	• None	• E-Mail • Messaging Program • Telephone • Video	• AR, DS, E, L, P, PS, T
• Nelson and Velasquez • United States • 2011	• Qualitative: General Description, Viewpoint, Discussion, Qualitative Analysis, or Non-Structured Review	• Psychology	• Licensed Practitioner	• None	• Video	• AC, AR, DS, E, L, PS, T-E
• Newby et al. • Australia • 2021	• Qualitative: General Description, Viewpoint, Discussion, Qualitative Analysis, or Non-Structured Review	• Psychiatry • Psychology	• Licensed Practitioner	• None	• Web-Based Assessment or Intervention	• AE, AI, PS
• Noronha et al. • The Netherlands • United States • 2022	• Qualitative: General Description, Viewpoint, Discussion, Qualitative Analysis, or Non-Structured Review	• None	• Graduate Level Trainee/Student • Licensed Practitioner • Resident, Intern, Fellow	• None	• Video	• AC, DEI, E, PS, T-E
• Panos et al. • United States • 2002	• Qualitative: General Description, Viewpoint, Discussion, Qualitative Analysis, or Non-Structured Review	• Social Work	• Graduate Level Trainee/Student	• None	• Video	• AR, DEI, DS, E, L
• Parish et al. • United States • 2021	• Qualitative: Program- Specific Outline or Discussion	• Counseling • Marriage/Family Therapy • Nursing • Nurse Practitioner • Psychiatry • Social Work	• Licensed Practitioner	• None	• App • E-Mail • Messaging Program • Telephone • Video	• DS, L, PS
• Patel et al. • United States • 2021	• Qualitative: General Description, Viewpoint, Discussion, Qualitative Analysis, or Non-Structured Review	• Psychology	• Graduate Level Trainee/Student • Licensed Practitioner	• None	• Video	• AA, DS, E, L/T, L, PS
• Perle • United States • 2020	• Mixed Method: Pre-Post Test or Multiple Time Points Survey or Assessment, and Qualitative Output	• Psychology	• Graduate Level Trainee/Student	• School/Academic	• App • Artificial Intelligence • E-Mail • Messaging Program • Nanomachine • Robotic • Social Media • Telephone • Video • Video Game • VR, AR, XR • Wearable • Web-Based Assessment or Intervention	• AR, DEI, DS, E, L, T-E
• Perle et al. • United States • 2022	• Quantitative: One-Time Survey or Assessment	• Psychology	• Graduate Level Trainee/Student	• School/Academic	• App • E-Mail • Messaging Program • Telephone • Video	• DS, E, L
• Perle et al. • United States • 2023	• Quantitative: One-Time Survey or Assessment	• Psychology	• Graduate Level Trainee/Student • Resident, Intern, Fellow	• None	• App • E-Mail • Messaging Program • Telephone • Video	• AA, AR, DS, E, L, T-E
• Phillips et al. • United States • 2021	• Qualitative: General Description, Viewpoint, Discussion, Qualitative Analysis, or Non-Structured Review	• Psychology	• Graduate Level Trainee/Student • Resident, Intern, Fellow	• School/Academic	• Video	• DEI, E, L/T
• Prabhakar • United States • 2013	• Qualitative: General Description, Viewpoint, Discussion, Qualitative Analysis, or Non-Structured Review	• Counseling • Psychology	• Licensed Practitioner	• None	• E-Mail • Messaging Program • Telephone • Video	• AE, DEI, DS, E, L
• Qureshi et al. • Australia • Saudi Arabia • 2021	• Qualitative: General Description, Viewpoint, Discussion, Qualitative Analysis, or Non-Structured Review	• Assistive Technology • Audiology • Dietic/Nutrition • Nursing • Occupational Therapy • Orthotist • Physical Therapy • Psychology • Social Work • Speech Therapy	• Licensed Practitioner	• Home-Based • Medical Center	• None	• AE, DEI, DS, E, L, T-E, T
• Rabe • South Africa • 2022	• Qualitative: General Description, Viewpoint, Discussion, Qualitative Analysis, or Non-Structured Review	• None	• Licensed Practitioner	• Primary Care	• E-Mail • Telephone • Video	• AC, AE, DS, E, PS, T-E
• Reamer • United States • 2013	• Qualitative: General Description, Viewpoint, Discussion, Qualitative Analysis, or Non-Structured Review	• Social Work	• Licensed Practitioner	• None	• E-Mail • Messaging Program • Social Media • Telephone • Video • Web-Based Assessment or Intervention	• AR, DS, E, L, P
• Rees and Haythornthwaite • Australia • 2004	• Qualitative: General Description, Viewpoint, Discussion, Qualitative Analysis, or Non-Structured Review	• Psychology	• Licensed Practitioner	• None	• Video	• AC, E, L
• Rezai-Rad et al. • Iran • 2012	• Quantitative: Pre-Post Test or Multiple Time Points Survey or Assessment	• None	• None	• None	• E-Mail • Telephone	• DS
• Roth et al. • United States • 2021	• Qualitative: General Description, Viewpoint, Discussion, Qualitative Analysis, or Non-Structured Review	• None	• Licensed Practitioner	• None	• Video	• AA, AC, AR, E, P, PS, T-E, T
• Rutledge et al. • United States • 2017	• Qualitative: General Description, Viewpoint, Discussion, Qualitative Analysis, or Non-Structured Review	• Nurse Practitioner	• Graduate Level Trainee/Student	• School/Academic	• Video	• AR, DS, E, IC, L, T-E
• Rutledge et al. • United States • 2011	• Qualitative: Program-Specific Outline or Discussion	• Nurse Practitioner	• Graduate Level Trainee/Student	• None	• Social Media	• DS, T
• Sabin & Skimming • United States • 2015	• Qualitative: General Description, Viewpoint, Discussion, Qualitative Analysis, or Non-Structured Review	• Psychiatry	• Licensed Practitioner	• None	• Video	• AE, DEI, DS, E, L, P, PS, T-E
• Saeed et al. • United States • 2017	• Qualitative: General Description, Viewpoint, Discussion, Qualitative Analysis, or Non-Structured Review	• Psychiatry	• Resident, Intern, Fellow	• None	• Video	• AA, AE, AR, DEI, DS, E, L, PS
• Schwartz and Lonborg • United States • 2011	• Qualitative: General Description, Viewpoint, Discussion, Qualitative Analysis, or Non-Structured Review	• Psychology	• Licensed Practitioner	• None	• E-Mail • Telephone • Video	• DS, E, L
• Shandley et al. • Australia • 2011	• Qualitative: Program-Specific Outline or Discussion	• Psychology	• Resident, Intern, Fellow	• School/Academic	• E-Mail • Video	• AC
• Sherbersky et al. • Northern Ireland • United Kingdom • 2021	• Qualitative: General Description, Viewpoint, Discussion, Qualitative Analysis, or Non-Structured Review	• Marriage/Family Therapy	• Graduate Level Trainee/Student • Licensed Practitioner	• None	• App • Telephone • Video	• E, L/T
• Simpson et al. • Australia • 2014	• Quantitative: Pre-Post Test or Multiple Time Points Survey or Assessment	• Psychology	• Graduate Level Trainee/Student • Resident, Intern, Fellow	• School/Academic	• E-Mail • Telephone • Video	• AA, AC, DS, E
• Smith et al. • United States • 2023	• Qualitative: General Description, Viewpoint, Discussion, Qualitative Analysis, or Non-Structured Review	• Marriage/Family Therapy	• Licensed Practitioner	• None	• Telephone • Video	• AA, AC, AI, T-E
• Spelten et al. • Australia • 2021	• Qualitative: Review Article	• Dietic/Nutrition • Music Therapy • Nursing • Occupational Therapy • Oncology • Physical Therapy • Psychology • Social Work • Speech Therapy	• None	• None	• App • E-Mail • Telephone • Video • Web-Based Assessment or Intervention	• AR, E, L
• Stoll et al. • Switzerland • 2020	• Qualitative: Review Article	• Psychiatry • Psychology • Social Work	• None	• None	• E-Mail • Messaging Program • Social Media • Telephone • Video	• AC, AE, AI, AR, DS, E, L, P, PS
• Strowd et al. • Australia • United States • 2022	• Qualitative: General Description, Viewpoint, Discussion, Qualitative Analysis, or Non-Structured Review	• Neurology • Oncology	• Licensed Practitioner	• None	• Telephone • Video	• AA, AC, AE, DEI, T-E
• Sunderji et al. • Canada • 2015	• Qualitative: Review Article	• Psychiatry	• Resident, Intern, Fellow	• None	• Video	• AC, L, PS, T-E
• Taylor and Fuller • United States • 2021	• Qualitative: General Description, Viewpoint, Discussion, Qualitative Analysis, or Non-Structured Review	• Nursing	• Graduate Level Trainee/Student • Licensed Practitioner	• None	• App • E-Mail • Telephone • Video	• DS, L, T-E
• Townsend et al. • Canada • South Africa • 2019	• Qualitative: General Description, Viewpoint, Discussion, Qualitative Analysis, or Non-Structured Review	• None	• Licensed Practitioner	• None	• Telephone • Video • Wearable	• AA, DS, E
• Webb and Orwig • United States • 2015	• Qualitative: General Description, Viewpoint, Discussion, Qualitative Analysis, or Non-Structured Review	• Psychology	• Licensed Practitioner	• None	• E-Mail • Messaging Program • Telephone • Video • Web-Based Assessment or Intervention	• DS, E, L, T
• Weisenmuller and Luzier • United States • 2022	• Qualitative: General Description, Viewpoint, Discussion, Qualitative Analysis, or Non-Structured Review	• Psychology	• Graduate Level Trainee/Student • Licensed Practitioner • Resident, Intern, Fellow	• None	• None	• AA, AE, DS, E, L
• Yellowlees et al. • United States • 2012	• Qualitative: General Description, Viewpoint, Discussion, Qualitative Analysis, or Non-Structured Review	• Psychology	• Licensed Practitioner	• None	• VR, AR, XR	• AE, DS, E, L
• Zha et al. • United States • 2020	• Qualitative: General Description, Viewpoint, Discussion, Qualitative Analysis, or Non-Structured Review	• Neurology	• Resident, Intern, Fellow	• Medical Center	• Video	• AA, AC, AR, T-E
• Ziade et al. • Algeria • Bahrain • Egypt • Iraq • Jordan • Kuwait • Lebanon • Libya • Morocco • Palestine • Qatar • Saudi Arabia • Syria • Tunisia • United Arab Emirates • 2022	• Quantitative: Pre-Post Test or Multiple Time Points Survey or Assessment	• Rheumatology	• Licensed Practitioner	• None	• Telephone • Video	• AA, AE, AR, DS, E, L, T-E
• Zickuhr et al. • United States • 2023	• Qualitative: General Description, Viewpoint, Discussion, Qualitative Analysis, or Non-Structured Review	• Rheumatology	• Licensed Practitioner • Resident, Intern, Fellow	• None	• App • Messaging Program • Telephone • Video	• AA, AC, AE, DEI, E, L, PS

^a^ The table summarizes information that was clearly identifiable in the coded manuscripts. A lack of coding (i.e., “none” or not listed in a specific column) does not necessarily suggest that the manuscript and its information is not applicable to a wider audience than what it indicated in the current table.

^b^ AA = Adaptations of assessments (i.e., Detailing of changes or considerations for assessment processes in terms of delivery or interpretation when integrating technology with healthcare services; e.g., modifying methods, normative data for technology-driven administration); AC = Adaptations of communication (i.e., Detail of changes or considerations for communication processes in terms of delivery or interpretation when integrating technology with healthcare services; e.g., modification of verbal communication, consideration of nonverbal communication); AE = Appropriateness Evaluation (i.e., Detailing of changes or considerations for evaluating whom technology is optimal or less optimal for when integrating technology with healthcare services; e.g., patient-specific factors or pathology); AI = Adaptations of interventions (i.e., Detailing of changes or considerations for intervention processes in terms of delivery or interpretation when integrating technology with healthcare services; e.g., adapting in-person methods for digital administration); AR = Administrative responsibilities (i.e., Detailing of changes or considerations for administrative responsibilities when integrating technology with healthcare services; e.g., documentation, billing, quality improvement analyses); DS = Data security (i.e., Detailing of changes or considerations for data security when integrating technology with healthcare services; e.g., encryption, passwords, technology destruction); DEI = Diversity, Equity, Inclusion (i.e., Detailing of diversity, equity, and/or inclusion when integrating technology with healthcare services; e.g., considering the role of race or ethnicity in the use of technology in clinical services); E = Ethics (i.e., Detailing of changes or considerations for ethics when integrating technology with healthcare services; e.g., ethical guidebooks, informed consent practices); IC = Interprofessional communication (i.e., Detailing of changes or considerations for interprofessional communication when integrating technology with healthcare services; e.g., methods of effectively sharing electronic patient data); L/T = Learning/Teaching of Others (i.e., Detailing of changes or considerations for educating others in the use of technology when integrated with healthcare services; e.g., methods of training in graduate or continuing education, methods of supervision); L = Legal (i.e., Detailing of changes or considerations for legality when integrating technology with healthcare services; e.g., interjurisdictional practice); PS = Patient safety (i.e., Detailing of changes or considerations for ensuring patient safety when integrating technology with healthcare services; e.g., safety plans); P = Professionalism (i.e., Detailing of changes or considerations for professionalism when integrating technology with healthcare services; e.g., professional boundaries); R = Research (i.e., Detailing of changes or considerations for research practices when integrating technology; e.g., influence of technology on self-tracking); SC = Self-care (i.e., Detailing of changes or considerations for self-care practices when integrating technology with healthcare services; e.g., ocular or muscular-skeletal adjustments to foster healthy use of technology); T-E = Techno-etiquette (i.e., Detailing of changes or considerations for techno-etiquette when integrating technology with healthcare services; e.g., technology selection processes, environment set-up, telepresence); T Troubleshooting (i.e., Detailing of changes or considerations for troubleshooting of technology when integrating technology with healthcare services; e.g., methods of self-addressing of technological issues, whom to contact to address technological issues).

### Summary findings

#### Paper type

Among included manuscripts (*N =* 109), 14 (12.8%) were coded as quantitative, 91 (83.5%) were coded as qualitative, and 4 (3.7%) were coded as mixed method. Among the 14 quantitative manuscripts, 6 (42.9%), were coded as a general one-time survey or assessment, and 8 (57.1%) were coded as a pre-post test or multiple time points survey or assessment. Among the 91 qualitative manuscripts, 71 (78.0%) were coded as a general description, viewpoint, discussion, qualitative analysis, or nonsystematic review; 10 (11.0%) were coded as a program-specific outline or discussion; and 10 (11.0%) were coded as a review article (e.g., formal systematic, scoping, or narrative review with a database search, goals, and/or search terms). Among the 4 mixed-method manuscripts, 1 (25.0%) was coded as a one-time survey or assessment and qualitative output, while 3 (75.0%) were coded as a pre-post test or multiple time points survey or assessment and qualitative output.

#### Publication date

All included manuscripts (*N =* 109) were published between 2000 and 2023, with a substantially greater number of publications per year in or following 2020 as compared to 2019 and earlier.

#### Author location

Among included manuscripts (*N =* 109), most authors had affiliations within the United States (86, 78.9%).

#### Specialty area

Specialty area of manuscript discussions (*N =* 109) varied widely across both mental health and medical domains. Psychology- (50, 45.9%), psychiatry- (28, 25.7%), and social work-focused manuscripts (21, 19.3%) were the three most discussed types of specialty areas.

#### Interdisciplinary discussion

Among included manuscripts (*N* = 109), 22 (20.2%) included more than one specialty as a focus of discussions.

#### Career stage

Of the total manuscripts (*N =* 109), 75 (68.8%) focused discussions on licensed practitioners, 34 (31.2%) focused on graduate level students/trainees, 28 (25.7%) focused on residents, interns, or fellows, one (0.9%) focused on paraprofessionals, and one (0.9%) focused on non-professional health operators.

#### Location of discussion

Of the total manuscripts (*N =* 109)*,* 15 (13.8%) focused on school/academic locations, 6 (5.5%) focused on medical centers, 2 (1.8%) focused on primary care clinics, and 1 (0.9% each) focused on college counseling, home-based, university outpatient clinic, and Veterans Affairs.

#### Types of technology

Of the total manuscripts (*N =* 109)*,* video (91, 83.5%), telephone (62, 56.9%), and email (44, 40.4%) were the three most discussed types of technology.

#### Identified competencies

Of the total manuscripts (*N =* 109*),* ethics (84, 77.1%), legal considerations (75, 68.8%), and data security (71, 65.1%) were the three most discussed types of competencies.

#### Additional considerations

Many excluded manuscripts focused on: (a) applications of technology in general healthcare service without discussion of competencies or training, (b) methods of training healthcare skills through the use of technology (e.g., e-learning), (c) practitioner or patient attitudes towards technology, (d) satisfaction with technology use, and (e) programmatic descriptions of technology integration with general healthcare clinics without detailing the applied competencies. Across studies with diverse focuses, authors consistently emphasized the necessity for more extensive training in both graduate education and professional practice to foster a comprehensive understanding and appreciation of the various competencies required for effective technology-enhanced practice.

In addition to the majority of included manuscripts focusing on mental health-focused specialties, it was recognized that among manuscripts not included, several other specialties have discussed or been discussed to utilize technology-enhanced practices, such as anesthesiology. Settings among manuscript not included, yet discussed as integrating technology, were also highly variable and included: childcare center, community mental health clinic, federally-qualified healthcare center, government agency (e.g., Department of Defense), mobile unit, prison/corrections, private practice, and military.

## Discussion

The current scoping review is believed to be the first to consolidate literature from across healthcare specialties to clarify competencies relevant to technology-enhanced practices. Among the 109 included manuscripts, all were published since 2000, with the majority being published during or post 2020. While the current study did not evaluate reasons for this finding, it was hypothesized that since a significant portion of the included manuscripts focused on telecommunication technologies, the increase in telehealth utilization post-COVID-19, combined with technology becoming more readily available and applied (12, 13), led to an increased recognition of the importance of competencies related to technology, thus fostering increased publication of study-relevant literature.

Related to the competencies themselves, as well as research question 1, literature discussed numerous modalities ranging from telecommunication technologies (e.g., video, telephone) to more esoteric technologies (e.g., wearable, VR/AR/XR). Findings not only suggested ongoing expansion of novel technologies into healthcare services, but also growing abilities of healthcare practitioners to harness the technologies to overcome historical barriers precluding effective healthcare. As one example, the use of wearable technologies permits ongoing physiological monitoring without relying on a patient to track their progress on paper-and-pencil forms, allowing for live and more accurate progress monitoring.

Related to research question 2, despite a multitude of articles suggesting the importance of developing competencies for the various technologies, relatively few highlighted specific competencies needed for different technologies. Fewer yet (*N* = 109) included a basic definition or operationalization of competencies to guide practitioners in the specifics of what to learn and how to adapt the technologies in order to effectively integrate them into their day-to-day practices. Additionally, among those detailing the competencies, the majority focused on ethical and legal considerations, as well as data security, with significant variability among the remaining competencies. Although specific reasons for why these three emerged as the most common are not currently clear, since the majority of the manuscripts included focused more on telehealth-related competencies (i.e., video, telephone) relative to other technologies (e.g., virtual reality, robotics), it was hypothesized that the marked increase in telehealth-related work following COVID-19 (12, 13) that coincided with the increased focus in ethical and legal practices emphasized by governing organizations (e.g., American Medical Association, American Psychiatric Association, American Psychological Association, National Association of Social Workers), licensing boards, and researchers, led to an increased recognition of the importance of ethics, legal, and data security specifically above and beyond any other possible competencies.

Finally, while some quantitative studies were reviewed, the majority of included publications were qualitative, and generally comprised of reviews, descriptions, viewpoints, recommendations, or program-specific overviews. As a result, it becomes clear that additional study is required to not only test often-suggested recommendations to better clarify what competencies are required as varying by technology, location, and specialty, but also how to best teach/acquire such information.

While not a primary target of the current study, some additional interesting findings were recognized. The review concluded that although a wider range of specialties than what was included in the final analysis were suggested to utilize technology in practice, discussions of competencies remain limited for many of these specialties. This becomes especially impactful for more specialized practitioners who may not seek cross-discipline journal articles to inform their practice, thus potentially missing relevant technology-related literature. For instance, although multiple manuscripts discussed the application of robotics (e.g., surgery), few manuscripts included in the final review discussed relevant competencies or means to gain such knowledge for the use of robotics in healthcare services.

### Integration and clinical application – iTECH model

Upon review of the findings, it became apparent that due to fragmentation and variability, no singular discussion was applicable to all specialties or technologies, or comprehensive enough to cover the wide range of possible service variations that may present for healthcare practitioners. To address this challenge, findings were organized into domains of competency to create the Intersectional Technology Education and Competency in Healthcare (iTECH) Model: a model designed as a comprehensive, intersectional, versatile, and multiprofessional means to guide practitioner education and training to foster optimal use of technologies in healthcare-related practices. The model is not only believed to influence educational and training activities, but also foster improved patient outcomes and practitioner effectiveness through guiding practitioners to the most pertinent competencies relative for their unique healthcare service.

**Figure 1:**
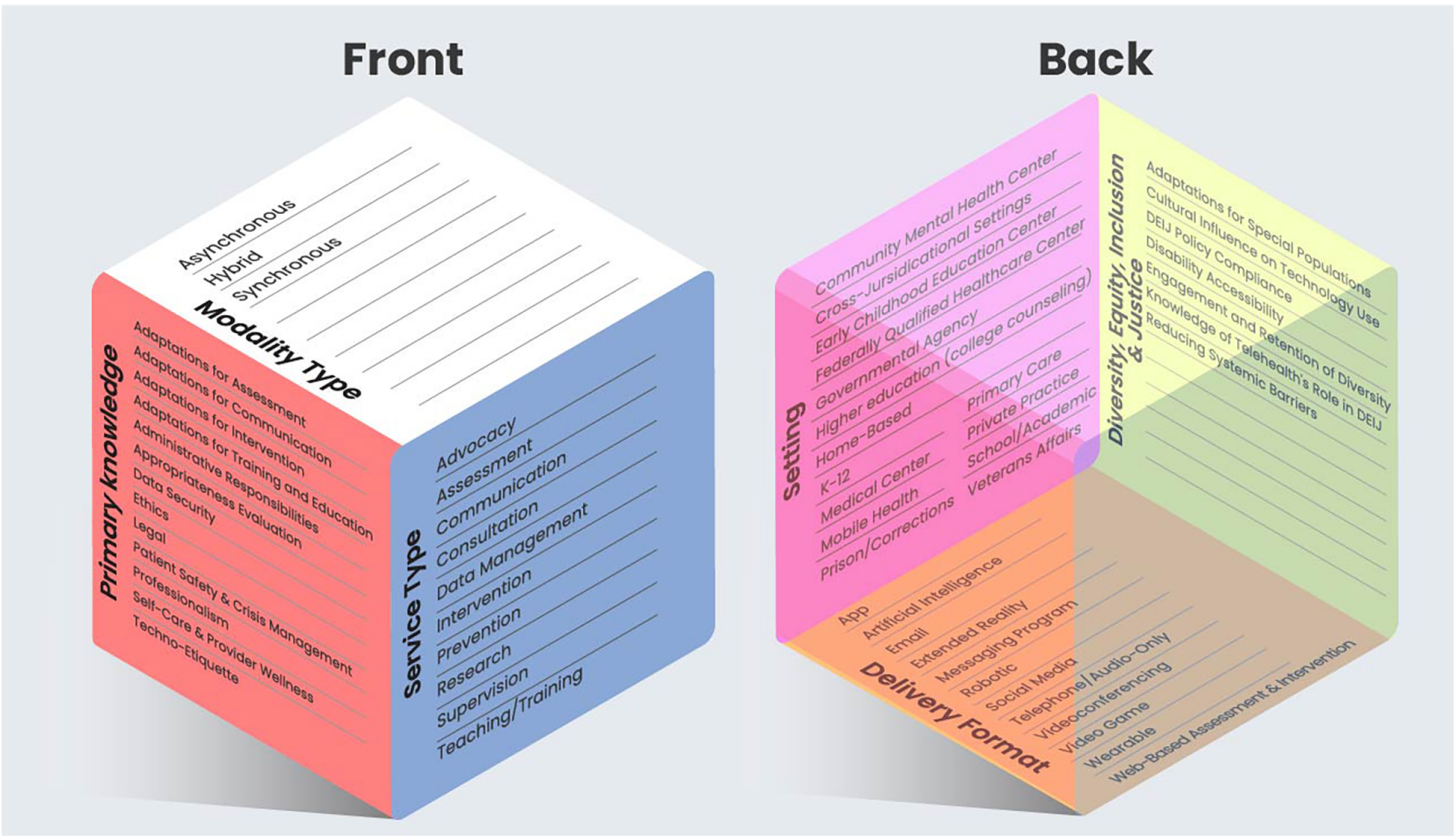
Intersectional technology education and competency in healthcare (iTECH) model^. a^Modality Type = The method in which technology is applied; Service Type = How the technology is used; Primary Knowledge = The type of technology-focused information practitioners should know to ensure ethical, legal, evidence-informed, and safe technology-enhanced practices; Delivery Format = The type of technology utilized; Setting = The locations in which the technology is utilized; Diversity, Equity, Inclusion, and Justice (DEIJ) = Consideration of factors relevant to the use of, and attitudes towards, the use of technology. ^b^The model can be adapted and applied for emerging technologies, settings, and uses.

Creation of the model was a multi-step process involving integration of study outcomes in combination with author consensus for grouping and naming. This approach aligned with past methodologies for telehealth/technology competency scoping reviews and model creation (26). In this way, information gathered from the study characteristics outlined in Table 2 (i.e., identified competencies, technology type, setting, career state focus, specialty area) created the foundation of domains for the novel model. Authors then combined their individual and collective experiences in training, research (including knowledge of the current study's non-included review articles), and professional work related to technology-enhanced practices to supplement the core information. Through this method, a domain for the model was established when consensus was reached that the identified domain was not only directly applicable to a healthcare practitioner's technology-enhanced practice, but provided a meaningful distinction from other domains, even if one influences another (26). This method (Figure 1) yielded six broad domains: (1) primary knowledge; (2) service type; (3) modality type; (4) delivery format; (5) setting; and (6) diversity, equity, inclusion, and justice (DEIJ). A seventh domain was also identified; however, based on author review of the literature, this domain, titled “supplemental knowledge,” was believed to be informative for practitioners, but nonessential (e.g., history of technology use). Each included domain is believed to be equally important to consider for any technology-enhanced practice, and can influence the others. For example, modality type can influence the types of services that could be provided, as well as the primary knowledge considerations required for effective use of that technology. While aspirational in nature, the model can be viewed as a means to guide an ethical, legal, evidence-informed, and safe practice through the selection of relevant competencies, while removing those less relevant to one's unique role. The healthcare practitioner can then utilize relevant competencies to focus readings, trainings, consultation, or other methods of gaining knowledge on these specific targets.

As an example of model application, consider a hospital-based child psychiatrist wanting to utilize an AI chatbot that tracks daily patient mood and patient-reported skill use. They may begin their application of the model by first clarifying the AI technology as their *delivery format*, with the *service type* focusing on assessment (i.e., data collection), the *modality type* as asynchronous, and the *setting type* as both a hospital (for practitioner) and home-based (for patient). The psychiatrist can use this information to educate themselves on *primary knowledge* relevant topics, including, but not limited to, differences between pencil-and-paper tracking vs. digital methods in terms of outcomes, data security considerations of sending and receiving patient data, and safety planning should a mood-related crisis arise. Additionally, the psychiatrist can explore setting-specific guidelines/requirements/restrictions of their organization for the use of AI. Finally, *DEIJ factors*, such as the role of systemic barriers and means of engagement and retention, should be considered, including the potential for financial-related data limitations (i.e., data allotment by plan) and how that can affect ongoing use of the chatbot. Additionally, biases (e.g., language) that could be introduced through the machine learning and natural language processing developmental operations of the AI chatbot should be considered (49).

### Limitations

Study findings should be interpreted within the context of recognized limitations. First, similar to other scoping reviews, literature may have been missed or omitted due to database selection, language criteria, and Boolean operators not matching all relevant manuscript meta data (50). Additionally, although the sequential adding of search terminology aligned with past literature and use of Rayyan, it is recognized that this could have resulted in some relevant literature being excluded due to not meeting full search term criteria for further review. Similarly, while published research procedures were followed, a screening of title and abstract may have inadvertently removed some literature that would have been relevant, but not clearly indicated as such in the title or abstract information. While attempts were made to control for human error (e.g., multiple coders, spot checking), given the large amount of data and subjective nature of the coding, human error cannot be fully ruled-out. Nevertheless, overall reporting is believed representative of the constructs within the literature. Although a decision was made to exclude non-peer-reviewed outlets (e.g., certificate programs), some non-studied outlets may include competency-related information not accounted for in peer-reviewed literature. Additionally, the lack of grey literature imposes a publication bias. Finally, in line with other scoping review methodologies (51, 52), the study was descriptive in nature and did not include an appraisal of the quality of the literature.

### Future directions

Future work related to the current study should include more databases and literature that were published following the current study's review period in order to determine any subsequent developments in technology-enhanced practice competencies. Additionally, a wider scope should be considered, including both peer-reviewed and non-peer-reviewed literature. For example, organization guidebooks (e.g., American Psychological Association, American Telemedicine Association), certificate programs, books, book chapters, and grey literature can be considered for inclusion. Future work should also seek to create universally-accepted standards for competency acquisition for technology-enhanced practices. More specifically, tighter operational definitions of a skill, testing and refinement, and proximal and distal (i.e., longitudinal) evaluation should be established. Standardization and long-term review can foster more objective measurement to evaluate successful methodologies, as well as areas for improvement. Additionally, standardization can allow for more direct evaluation of relevant outcome measures, such as cost-benefit assessments for the individual, the organization, and the patient in terms of positive outcomes and financial costs. Finally, future work should seek to further explore the iTECH model through two means. First, the model should be compared to other known models/recommendations of technology-related competency acquisition by guiding organizations, including the American Medical Association (24), the American Psychological Association (22), American Psychiatric Association (23), American Telemedicine Association (21), World Health Organization (9), and the American medical Informatics Association (53), as well as researcher-based models/recommendations [e.g., (26)]. This comparison can allow for identification of strengths and areas of improvement for the iTECH model. Second, it is essential that the model is implemented and assessed in healthcare practitioner's training curriculum to determine its influence in fostering both knowledge and hands-on competencies. This evaluation can consider usability and adaptability to different healthcare specialties and technologies. Pre- and post-education assessment can clarify trainer attitudes towards the model, trainee attitudes towards the model, and educational outcomes in terms of both evidence-informed understanding of utilized technologies and application. Once determined useful, the model can serve as a guide to create technology-enhanced practice curriculum for training programs in terms of coursework, applied hands-on work, and supervision. As the model is implemented, it is important to utilize an established framework for skill acquisition and education. One recommended method is the Kirkpatrick Model (54), as this model has been heavily cited within the literature for such purposes (55, 56). This model focuses on four levels of evaluation: reaction, learning, behavior, and results. Reactions focus on how trainees like a particular training model. Such an evaluation could include both quantitative (e.g., surveys/ratings of satisfaction) and/or qualitative (e.g., focus groups) assessments to measure trainees' perceptions of the model (54, 55). Effective learning assessment measures both program acceptance and knowledge transfer while gathering feedback to enhance future training. When trainees view a program positively, they are more likely to engage with and retain the material (54). Of important note, it is essential that evaluation objectively measures the amount of learning that takes place in addition to subjective experiences. Such evaluations can be completed through performance testing, simulations, case studies, and pre to post assessments (55). Behavior evaluates real-world behavior change with comparison of an intervention group to a control group. According to Kirkpatrick (54), this approach demands a scientific methodology using systematic before-and-after performance evaluations (examining both proximal and distal outcomes) with statistical analyses to measure behavioral changes. Finally, results evaluate system-wide or organizational impacts of the training program, such as improved evidence-informed practices, reduced costs, higher quality, increased production rates of satisfaction, varying based on specific program goals (54, 55). Graduate education presents an optimal opportunity to integrate the iTECH model with Kirkpatrick's method, enabling evaluation of technology-enhanced practices by training directors across various levels including practicum, internship, fellowship, and residency programs, depending on the healthcare specialty. Following evaluation, the iTECH model's implementation can be modified through an iterative approach to implementation. As trainee's advance through their training, milestone levels can be evaluated through the Dreyfus and Dreyfus model (57, 58), which evaluates the acquisition of expertise as a developmental process through five primary steps: novice, to advanced beginner, competent, proficient, and expert. As adapted by Hilty et al. (55), novice could be equated to a graduate student, advanced beginner to a first-year resident, competent to a senior resident, proficient to a graduating resident, and expert as a competent and licensed practitioner.

Utilizing such an educational framework, integration of the iTECH model can occur at multiple stages of one's professional development to account for the need for both didactic and hands-on experiential training (11). First, didactic information regarding relevant technologies can be provided during or following the introduction of general healthcare strategies. More specifically, the application of healthcare techniques (e.g., assessment, interventions) can be discussed in terms of both traditional and technology-enhanced methods. Such discussion can focus on general use of the technologies, relevant research, and both benefits and limitations of usage relative to non-technology methods in order to foster critical thinking of the use of the technologies (11). For example, a course describing ethical and legal healthcare can also include a discussion of jurisdictional practices and differences when implementing video or robotics (e.g., surgery) that may span different states, provinces, territories, or countries. Methods of learning about such differences, as well as how to safely account for and navigate such differences can also be outlined. Supplementing general discussions, advanced coursework can be created for either the broad integration of technology, or for specific technologies, such as a class on robotics for surgery, artificial intelligence in the use of medical research, or video for psychotherapy. While limited discussions of such coursework are available in the literature, and predominantly focus on telehealth rather than other technologies, courses and curriculums that can provide templates from which additional technology-focused work can be derived include Perle's Introduction to Telehealth for Clinical Psychologists (59), the University of Illinois College of Medicine at Peoria's robotic surgery training curriculum (60), and Greenberg and colleague's description of a pilot robotic surgery curriculum (61). Following the trainees acquisition of didactic information, hands-on experiences should be completed with classroom-based role play and simulation labs, as well as through real-world application in placements (e.g., practicum, internship, fellowship, residency). All work should be supervised with scheduled and/or live supervision, as appropriate to the site and training model. Technology-focused supervision must not only include consideration of the general healthcare practices and patient outcomes, but also numerous technology-focused considerations. For instance, supervision should include discussions of how the technologies were used, how they compared to non-technology-enhanced methods, the benefits of the integration of the technologies, limitations of the technologies, how any arising issues were addressed, how the technologies were perceived by the practitioner, how the technologies were perceived by the patient, and how the technologies interacted to change the healthcare service being provided. Training and supervision requirements can be guided by principles and standards set by governing organizations of the healthcare specialty, such as the American Psychological Association's Commission on Accreditation (62) and the Accreditation Council for Graduate Medical Education (63, 64). To monitor the impact of the novel curriculum and methodologies, graduate programs should implement both objective and subjective methods of assessment outside of the coursework and fieldwork. Assessment can include measurement of the improvement in both knowledge and hands-on technology-enhanced competencies as defined by the iTECH model and available literature, as well as aligning standards set forth by accreditation governance. Assessment can also evaluate attitude changes towards technologies, perceived ease of use of the technologies, perceived benefits and limitations of the technologies, supervisor challenges with teaching technologies, and trainee challenges with learning the technologies. Finally, distal assessment should evaluate if the training fostered ongoing use of technologies, as well as which types, into the future (11). Given rapid developments, ongoing continuing education post graduate education is essential. As a result, training institutions (e.g., universities, hospitals, licensing boards, professional organizations) must design new self-guided and professionally-led continuing education series to foster ongoing education of specific technologies for specific healthcare specialties. Such strategies can include self-education through continuing education literature, didactic presentations either live or via webinar, and hands-on training workshops. Well-rounded training and knowledge are believed necessary to foster optimal technology-enhanced healthcare services that adapt as the field continues to evolve to yield new technologies and competencies. Education is not only to ensure a practitioner's ability to effectively integrate and maintain the technologies, but also to ensure that practitioners are equipped with research-informed rationales for what technology works best for who, as well as which may be contraindicated or to be used with caution. Further, as not all individuals may equally respond to different technologies and strategies, methods of how to adapt the technologies for unique population demographics (e.g., age, education, race, language, disability, socioeconomic status, technology comfort level) and pathologies are essential.

## Conclusions

The current scoping review suggested ongoing expansion of technology into healthcare practices, indicating the need for greater practitioner resources to ensure their acquisition of necessary competencies to foster ethical, legal, evidence-informed and safe practices. In doing so, practitioners can acquire necessary knowledge to be able to tailor technologies and clinical services to unique services, population demographics, and pathologies. Nevertheless, findings also indicated that few peer-reviewed manuscripts highlighted and expounded upon specific competencies needed or recommended for practice. Additionally, despite the review yielding a variety of technologies used across healthcare specialization, the review process allowed for recognition that a wider range of technologies are believed to be utilized across a larger scope of healthcare specialties than what was recognized in the final scoping review as based upon the inclusionary criteria (e.g., many studies did not expound upon competencies), necessitating the need for greater competency development and dissemination to inform practitioners. To address this gap, the iTECH Model was created to guide a practitioner's technology-enhanced practice. The model is believed to assist practitioners in identifying relevant competencies to ensure knowledge and hands-on experiences relevant to their unique practices in order to foster optimal care and patient outcomes, while reducing possible issues. Although believed helpful, the current work is viewed as a first-step. There remains a need for additional study to not only better understand literature-suggested competencies, as evolving over time, but also to explore best methods for integrating the iTECH model into graduate coursework, real-world experiences, and continuing education to enhance its utility for diverse healthcare practitioners.
