# Supplementary material for: Technology-enhanced practice competencies: scoping review and novel model development
Source: Front Digit Health. 2025 Apr 25;7:1571518. doi: 10.3389/fdgth.2025.1571518 (PMC12061994; doi:10.3389/fdgth.2025.1571518)
Supplement: Supplementary file 1 [file Datasheet1.pdf]

**Supplementary Table 1.** Search Process Boolean Operators

(tele OR e- OR internet OR online OR distance OR web-based OR digital OR video OR phone OR app-based OR app OR email OR text OR virtual reality OR VR OR artificial intelligence OR AI or augmented reality OR AR or nanomachines OR robotics OR robot OR technology OR telebehavioral health)

AND

(care OR therapy OR health OR medicine OR intervention OR assessment OR consultation OR coaching)

AND

(guidelines OR guide OR guidebook OR recommendations OR competency OR competencies OR model OR training OR education)

**Supplementary Table 2.** Screening Terms

| | | | | | |
|---|---|---|---|---|---|
| **Screening Terms 1: Education-, teaching-, and training-related** | | | | | |
| Competency | Training | Education | Learning | Proficiency | Skill |
| Teaching | Guidance | Guide | Model | Framework | Blueprint |
| Best practice | Curriculum | Curricula | Residency | Internship | Fellowship |
| Target | Goal | | | | |
| | | | | | |
| **Screening Terms 2: Technology-related** | | | | | |
| Tele | Tele- | Telehealth | Telecare | Telemedicine | Teletherapy |
| Telepsychology | Teleintervention | Teleassessment | Telebehavioral | Tech | Tech- |
| Technology | e- | ehealth | ecare | emedicine | etherapy |
| epsychology | eintervention | eassessment | Internet | Internet- | Online |
| Online- | Digital | Digital- | Distance | Distance- | Mobile |
| Mobile- | mhealth | mhealth- | m- | Web | Web- |
| Virtual | Virtual- | | | | |
| | | | | | |
| **Screening Terms 3: Healthcare-related** [a] | | | | | |
| Provider | Clinician | Practitioner | Doctor | Physician | Healthcare |
| Medical | Psychological | Psychology | Hematology | Hematologist | ICU |
| Intensive Care | Psychiatry | Psychiatrist | Radiology | Radiologist | Endocrinology |
| Endocrinologist | Stroke | Pathology | Pathologist | Surgery | Surgeon |
| Obstetrics | OBGYN | Obstetrician | Pediatrics | Pediatrician | Oncology |
| Oncologist | Gynecology | Gynecologist | Neurology | Neurologist | Internal Medicine |
| Internist | Family Medicine | Anesthesiology | Anesthesiologist | Orthopedics | Orthopedic |
| Otorhinolaryngology | Otorhinolaryngologist | Dermatology | Dermatologist | Ophthalmology | Ophthalmologist |

| | | | | | |
|---|---|---|---|---|---|
| Cardiology | Cardiologist | Urology | Urologist | Physical Therapy | Physical Therapist |
| OB/GYN | Occupational Therapy | Occupational Therapist | Gastroenterology | Gastroenterologist | Immunology |
| Immunologist | Geriatrics | Gerontology | Nephrology | Nephrologist | Rheumatology |
| Rheumatologist | Pulmonology | Pulmonologist | Neonatology | Neonatologist | Genetics |
| Geneticist | Sleep Medicine | Burn | Nurse | Nursing | Social Work |
| Social Worker | Counseling | Counselor | Speech Therapy | Speech Therapist | Neuropsychology |
| Neuropsychologist | Psychologist | Physician Assistant | PA | Nurse Practitioner | NP |
| RN | ENT | Otolaryngology | Otolaryngologist | Medicine | Therapist |
| Therapy | PT | OT | | | |
| Teleprovider | Tele-provider | Teleclinician | Tele-clinician | Telepractitioner | Tele-practitioner |
| Teledoctor | Tele-doctor | Telephysician | Tele-physician | Telehealthcare | Tele-healthcare |
| Telemedical | Tele-medical | Telepsychological | Tele-psychological | Telepsychology | Tele-psychology |
| Telehematology | Tele-hematology | Telehematologist | Tele-hematologist | TeleICU | Tele-ICU |
| Teleintensive care | Tele-intensive care | Telepsychiatry | Tele-psychiatry | Telepsychiatrist | Tele-psychiatrist |
| Teleradiology | Tele-radiology | Teleradiologist | Tele-radiologist | Teleendocrinology | Tele-endocrinology |
| Teleendocrinologist | Tele-endocrinologist | Telestroke | Tele-stroke | Telepathology | Tele-pathology |
| Telepathologist | Tele-pathologist | Telesurgery | Tele-surgery | Telesurgeon | Tele-surgeon |
| Teleobstetrics | Tele-obstetrics | Teleobgyn | Tele-obgyn | Teleobstetrician | Tele-obstetrician |
| Telepediatrics | Tele-pediatrics | Telepediatrician | Tele-pediatrician | Teleoncology | Tele-oncology |
| Teleoncologist | Tele-oncologist | Telegynecology | Tele-gynecology | Telegynecologist | Tele-gynecologist |
| Teleneurology | Tele-neurology | Teleneurologist | Tele-neurologist | Teleinternal medicine | Tele-internal medicine |

| | | | | | |
|---|---|---|---|---|---|
| Teleinternist | Tele-internist | Telefamily medicine | Tele-family medicine | Teleanesthesiology | Tele-anesthesiology |
| Teleanesthesiologist | Tele-anesthesiologist | Teleorthopedics | Tele-orthopedics | Teleorthopedic | Tele-orthopedic |
| Teleotorhinolaryngology | Tele-otorhinolaryngology | Teleotorhinolaryngologist | Tele-otorhinolaryngologist | Teledermatology | Tele-dermatology |
| Teledermatologist | Tele-dermatologist | Teleophthalmology | Tele-ophthalmology | Teleophthalmologist | Tele-ophthalmologist |
| Telecardiology | Tele-cardiology | Telecardiologist | Tele-cardiologist | Teleurology | Tele-urology |
| Teleurologist | Tele-urologist | Telephysical therapy | Tele-physical therapy | Telephysical therapist | Tele-physical therapist |
| Teleob/gyn | Teleob/gyn | Teleoccupational therapy | Tele-occupational therapy | Teleoccupational therapist | Tele-occupational therapist |
| Telegastroenterology | Tele-gastroenterology | Telegastroenterologist | Tele-gastroenterologist | Teleimmunology | Tele-immunology |
| Teleimmunologist | Tele-immunologist | Telegeriatrics | Tele-geriatrics | Telegerontology | Tele-gerontology |
| Telenephrology | Tele-nephrology | Telenephrologist | Tele-nephrologist | Telerheumatology | Tele-rheumatology |
| Telerheumatologist | Tele-rheumatologist | Telepulmonology | Tele-pulmonology | Telepulmonologist | Tele-pulmonologist |
| Teleneonatology | Tele-neonatology | Teleneonatologist | Tele-neonatologist | Telegenetics | Tele-genetics |
| Telegeneticist | Tele-geneticist | Telesleep Medicine | Tele-sleep Medicine | Teleburn | Tele-burn |
| Telenurse | Tele-nurse | Telenursing | Tele-nursing | Telesocial Work | Tele-social Work |
| Telesocial Worker | Tele-social Worker | Telecounseling | Tele-counseling | Telecounselor | Tele-counselor |
| Telespeech Therapy | Tele-speech Therapy | Telespeech Therapist | Tele-speech Therapist | Teleneuropsychology | Tele-neuropsychology |

| | | | | | |
|---|---|---|---|---|---|
| Teleneuropsychologist | Tele-neuropsychologist | Telepsychologist | Tele-psychologist | Telephysician Assistant | Tele-physician Assistant |
| TelePA | Tele-PA | Telenurse practitioner | Tele-nurse practitioner | TeleNP | Tele-NP |
| TeleRN | Tele-RN | TeleENT | Tele-ENT | Teleotolaryngology | Tele-otolaryngology |
| Teleotolaryngologist | Tele-otolaryngologist | Telemedicine | Tele-medicine | Teletherapist | Tele-therapist |
| Teletherapy | Tele-therapy | TelePT | Tele-PT | TeleOT | Tele-OT |
| Telebehavioral | Tele-behavioral | | | | |

[a] Due to Rayyan keyword programming, tele was added to identified specialties to ensure carry over from screening stage 2 to 3 (e.g., psychology would not include telepsychology unless telepsychology was uniquely added).

**Supplementary Figure 1.** PRISMA Diagram

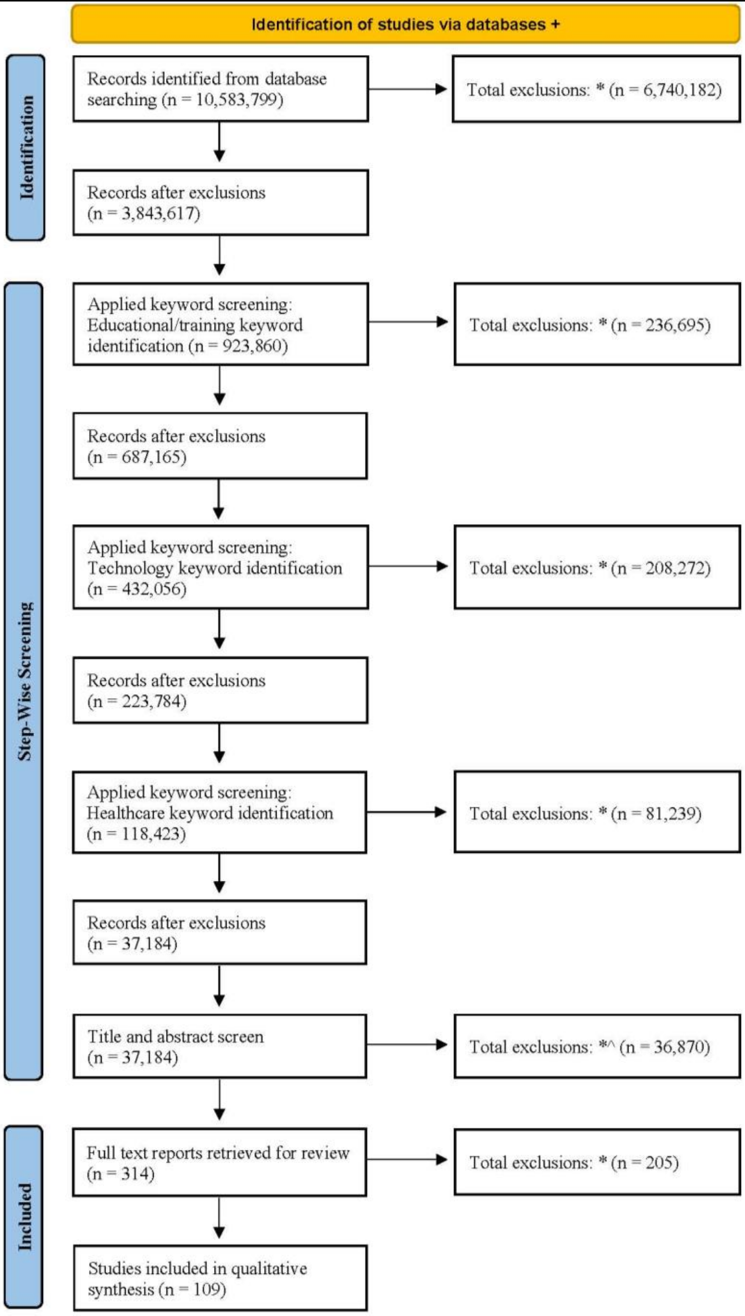


+Due to the review's large number of entries, a single database was unable to be used due to Rayyan's data/size limits. As a result, data was spread across multiple individual databases, precluding a singular review for all duplicates, languages other than English, etc. at any particular step. To account for this, data and exclusion were reported at each step in the review process. All duplicates and non-inclusionary documents were ultimately identified and excluded by the final steps.

*Exclusions included duplications and/or items not meeting inclusionary criteria

^One manuscript was unable to be secured via available resources.

## Scoping Review References


- Abbott, J-A.M., Klein, B., & Ciechomski, L. Best practices in online therapy. *J Technol Hum Serv.* (2008) 26(2-4):360-375. https://doi.org/10.1080/15228830802097257
- Alkureishi, M.A., et al. Teaching telemedicine: The next frontier for medical educators. *JMIR Med Educ.* (2021) 7(2):e29099. https://doi.org/10.2196/29099
- Almubark, B.M., et al. Telehealth clinical practice guide for occupational therapy, physical therapy, and speech and language pathology: A Saudi and middle eastern guide. *J Telemed Telecare.* (2022) 28(5):636-642. https://doi.org/10.1089/tmj.2021.0021
- Arends, R., et al. Enhancing the nurse practitioner curriculum to improve telehealth competency. *J Am Assoc Nurse Pract.* (2021) 33:391-397. https://doi.org/10.1097/JXX.0000000000000303
- Armstrong, C.M. Mobile health provider training: Results and lessons learned from year four of training on core competencies for mobile health in clinical care. *J Technol Behav Sci.* (2019) 4:86-92. https://doi.org/10.1007/s41347-019-00089-8
- Baker, D.C., & Bufka, L.F. Preparing for the telehealth world: Navigating legal, regulatory, reimbursement, and ethical issues in an electronic age. *Prof Psychol Res Pr.* (2011) 42(6):405-411. https://doi.org/10.1037/a0025037
- Baltimore, M.L. Ethical considerations in the use of technology for marriage and family counselors. *Fam J.* (2000) 8(4):390-393. https://doi.org/10.1177/1066480700084010
- Baumes, A., Colic, M., & Araiba, S. Comparison of telehealth-related ethics and guidelines and a checklist for ethical decision making in the midst of the COVID-19 pandemic. *Behav Anal Pract.* (2020) 13:736-747. https://doi.org/10.1007/s40617-020-00475-2
- Brimley, S., et al. The emerging critical role of telemedicine in the urology clinic: A practice guide. *Sex Med Rev.* (2021) 9:289-295. https://doi.org/10.1016/j.sxmr.2020.12.002
- Casline, E., et al. Considerations for assessment training competencies in health service psychology programs in the age of COVID-19. *Train Educ Prof Psychol.* (2021) 15(4):267-275. https://doi.org/10.1037/tep0000360
- Caver, K.A., et al. Telemental health training in the Veterans Administration Puget Sound Health Care System. *J Clin Psychol.* (2020) 76:1108-1124. https://doi.org/10.1002/jclp.22797
- Chike-Harris, K.E., et al. Telehealth professionalism education for advanced practice nursing students. *Nurs Educ Perspect.* (2022) 43(6):382-383. https://doi.org/10.1097/01.NEP.0000000000000910
- Chipps, J., Ramlall, S., & Mars, M. Practice guidelines for videoconferencing-based telepsychiatry in South Africa. *Afr J Psychiatry.* (2012) 15:271-282. https://doi.org/ 10.4314/ajpsy.v15i4.35
- Cooper, S.E., Campbell, J.F., & Barnwell, S.S. Telepsychology: A primer for counseling psychologists. *Couns Psychol.* (2019) 47(8):1074-1114. https://doi.org/10.1177/0011000019895276
- Costich, M., et al. Design and implementation of an interactive, competency-based pilot pediatric telemedicine curriculum. *Med Educ Online.* (2021) 26:1911019. https://doi.org/10.1080/10872981.2021.1911019

- Daniel, H., & Sulmasy, L.S. Policy recommendations to guide the use of telemedicine in primary care settings: An American College of Physicians position paper. *Ann Intern Med.* (2015) 163(10):787-789. https://doi.org/10.7326/M15-0498
- de Leo, G., et al. A virtual reality system for the training of volunteers involved in health emergency situations. *Cyberpsychol Behav.* (2023) 6(3):267-274. https://doi.org/10.1089/109493103322011155
- DeJong, S.M. Professionalism and adolescent psychiatry in the digital age. *Adolesc Psychiatry.* (2014) 4(2):64-72.
- DeJong, S.M., et al. Professionalism and the internet in psychiatry: What to teach and how to teach it. *Acad Psychiatry.* (2012) 36(5):356-362. https://doi.org/10.1176/appi.ap.11050097
- DeJong, C., Lucey, C.R., & Dudley, R.A. Incorporating a new technology while doing no harm, virtually. *JAMA.* (2015) 314(22):2351-2352. https://doi.org/10.1001/jama.2015.13572
- Dopp, A.R., et al. Incorporating telehealth into health service psychology training: A mixed-method study of student perspectives. *Digit Health.* (2021) 7:1-15. https://doi.org/10.1177/2055207620980222
- Drude, K.P., et al. Telebehavioral health competencies in interprofessional education and training: A pathway to interprofessional practice. *J Technol Behav Sci.* (2020) 5(1):30-39. https://doi.org/10.1007/s41347-019-00112-y
- Drum, K.B., & Littleton, H.L. Therapeutic boundaries in telepsychology: Unique issues and best practice recommendations. *Prof Psychol Res Pr.* (2014) 45(5):309-315. https://doi.org/10.1037/a0036127
- Farmer, R.L., et al. Teleassessment with children and adolescents during the coronavirus (COVID-19) pandemic and beyond: Practice and policy implications. *Prof Psychol Res Pr.* (2020) 51(5):477-487. https://doi.org/10.1037/pro0000349
- Fitzgerald, T.D., et al. Ethical and legal considerations for internet-based psychotherapy. *Cogn Behav Ther.* (2010) 39(3):173-187. https://doi.org/10.1080/16506071003636046
- Frankl, S.E., et al. Preparing future doctors for telemedicine: An asynchronous curriculum for medical students implemented during the COVID-19 pandemic. *Acad Med.* (2021) 96:1696-1701. https://doi.org/10.1097/ACM.0000000000004260
- Fuertes-Guiro, F, & Velasco, E.V. Ethical aspects involving the use of information technology in new surgical applications: Telesurgery and surgical telementoring. *Acta Bioeth.* (2018) 24(2):167-179.
- Gibson, N.A., Arends, R., & Hendrickx L. Tele-u to tele-icu: Telehealth nursing education. *Crit Care Nurse.* (2021) 41(5):34-40. https://doi.org/10.4037/ccn2021109
- Gifford, V., et al. Continuing education training focused on the development of behavioral telehealth competencies in behavioral healthcare providers. *Rural Remote Health.* (2012) 12(4):1-15.
- Govindarajan, R., et al. Developing an outline for teleneurology curriculum: AAN Telemedicine Work Group recommendations. *Neurol.* (2017) 89:951-959. https://doi.org/10.1212/WNL.0000000000004285
- Hames, J.L., et al. Navigating uncharted waters: Considerations for training clinics in the rapid transition to telepsychology and telesupervision during COVID-19. *J Psychother Integr.* (2020) 30(2):348-365. https://doi.org/10.1037/int0000224

- Hart, A., et al. Developing telemedicine curriculum competencies for graduate medical education: Outcomes of a modified delphi process. *Acad Med.* (2022) 97:577-585. https://doi.org/10.1097/ACM.0000000000004463
- Haydon, H.M., et al. Addressing concerns and adapting psychological techniques for videoconsultations: A practical guide. *Clin Psychol.* (2021) 25(2):179-186. https://doi.org/10.1080/13284207.2021.1916904
- Hertlein, K.M., et al. Toward proficiency in telebehavioral health: Applying interprofessional competencies in couple and family therapy. *J Marital Fam Ther.* (2021) 47:359-374. https://doi.org/10.1111/jmft.12496
- Hertlein, K.M., Drude, K., & Jordan, S.S. "What next?": Toward telebehavioral health sustainability in couple and family therapy. *J Marital Fam Ther.* (2021) 47:551-565. https://doi.org/10.1111/jmft.12510
- Hilty, D.M., et al. Mobile health, smartphone/device, and apps for psychiatry and medicine: Competencies, training, and faculty development issues. *Psychiatr Clin N Am.* (2019) 42:513-534. https://doi.org/10.1016/j.psc.2019.05.007
- Hilty, D.M., et al. A framework for competencies for the use of mobile technologies in psychiatry and medicine: Scoping review. *JMIR Ment Health.* (2020) 8(2):e12229. https://doi.org/10.2196/12229
- Hilty, D.M., et al. A framework for telepsychiatric training and e-health: Competency-based education, evaluation and implications. *Int Rev Psychiatry.* (2015) 27(6):569-592. https://doi.org/10.3109/09540261.2015.1091292
- Hilty, D.M., et al. Telebehavioral health, telemental health, e-therapy and e-health competencies: The need for an interprofessional framework. *J Technol Behav Sci.* (2017) 2:171-189. https://doi.org/10.1007/s41347-017-0036-0
- Hilty, D.M., et al. Telepsychiatry and other technologies for integrated care: Evidence base, best practice models and competencies. *Int Rev Psychiatry.* (2018) 30(6):292-309. https://doi.org/10.1080/09540261.2019.1571483
- Hilty, D.M., et al. A scoping review to develop a framework of asynchronous technology competencies for psychiatry and medicine. *J Technol Behav Sci.* (2021) 6:231-251. https://doi.org/10.1007/s41347-020-00185-0
- Hilty, D.M., et al. Child and adolescent asynchronous technology competencies for clinical care and training: Scoping review. *Fam Syst Health.* (2021) 39(1):121-152. https://doi.org/10.1037/fsh0000536
- Jagolino, A.L., et al. A call for formal telemedicine training during stroke fellowship. *Contemp Issues: Innov High Educ.* (2016) 86:1827-1833. https://doi.org/10.1212/WNL.0000000000002568
- Jarvis-Selinger, S., et al. Clinical telehealth across the disciplines: Lessons learned. *Telemed J e-Health.* (2008) 14(7):720-725. https://doi.org/10.1089/tmj.2007.0108
- Johnson, G.R. Toward uniform competency standards in telepsychology: A proposed framework for Canadian psychologists. *Can Psychol.* (2014) 55(4):291-302. https://doi.org/10.1037/a0038002
- Joint Task Force for the Development of Telepsychology Guidelines for Psychologists. Guidelines for the practice of telepsychology. *Am Psychol.* (2013) 68(9):791-800. https://doi.org/10.1037/a0035001

- Jones, R.M., Leonard, S., & Birmingham, L. Setting up a telepsychiatry service. *BJPsych Bul.* (2006);30:464-467. https://doi.org/10.1192/pb.30.12.464
- Karcher, N.R., & Presser, N.R. Ethical and legal issues addressing the use of mobile health (mHealth) as an adjunct to psychotherapy. *Ethics Behav.* (2016) 28(1):1-22. https://doi.org/10.1080/10508422.2016.1229187
- Keswani, A., Brooks, J.P., & Khoury, P. The future of telehealth in allergy and immunology training. *J Allergy Clin Immunol Pract.* (2020) 8(7):2135-2141. https://doi.org/10.1016/j.jaip.2020.05.009
- Khan, S., et al. A national pediatric telepsychiatry curriculum for graduate medical education and continuing medical education. *J Child Adolesc Psychopharmacol.* (2021) 31(7):457-463. https://doi.org/10.1089/cap.2021.0024
- Khan, S., & Ramtekkar, U. Child and adolescent telepsychiatry education and training. *Psychiatr Clin N Am*. (2019) 42:555-562. https://doi.org/10.1016/j.psc.2019.08.010
- Koh, S., et al. Psychiatrists' use of electronic communication and social media and a proposed framework for future guidelines. *J Psychiatr Pract.* (2013) 19:254-263. https://doi.org/10.1097/01.pra.0000430511.90509.e2
- Lockwood, M.M., et al. Telemedicine in adult rheumatology: In practice and in training. *Arthritis Care Res.* (2022) 74(8):1227-1233. https://doi.org/10.1002/acr.24569
- Loman, M., et al. "How to" operate a pediatric neuropsychology practice during the COVID-19 pandemic: Real tips from one practice's experience. *Child Neurpsychol.* (2021) 27(2):251-279. https://doi.org/10.1080/09297049.2020.1830962
- Lustgarten, S.D., & Elhai, J.D. Technology use in mental health practice and research: Legal and ethical risks. *Clin Psychol.* (2018) 25:e12234. https://doi.org/10.1111/cpsp.12234
- Maheu, M.M., et al. A framework of interprofessional telebehavioral health competencies: Implementation and challenges moving forward. *Acad Psychiatry.* (2018) 42:825-833. https://doi.org/10.1007/s40596-018-0988-1
- Maheu, M.M., et al. An interprofessional framework for telebehavioral health competencies. *J Technol Behav Sci.* (2017) 2:190-210. https://doi.org/10.1007/s41347-017-0038-y
- Maheu, M.M., et al. Correction to: An interprofessional framework for telebehavioral health competencies. *J Technol Behav Sci.* (2018) 3:108-140. https://doi.org/10.1007/s41347-018-0046-6
- Maheu, M.M., et al. Interprofessional telebehavioral health competencies framework: Implications for telepsychology. *Prof Psychol Res Pr.* (2021) 52(5):439-448. https://doi.org/10.1037/pro0000400
- Mallen, M.J., Vogel, D.L., & Rochlen, A.B. The practical aspects of online counseling: Ethics, training, technology, and competency. *Couns Psychol.* (2005) 33:776-818. https://doi.org/10.1177/0011000005278625
- Martin, J.N., Millan, F., & Campbell, L.F. Telepsychology practice: Primer and first steps. *Pract Innov.* (2020) 5(2):114-127. https://doi.org/10.1037/pri0000111
- McCord, C., et al. A consolidated model for telepsychology practice. *J Clin Psychol.* (2020) 76(6):1060-1082. https://doi.org/10.1002/jclp.22954

- McCord, C.E., et al. Training the next generation of counseling psychologists in the practice of telepsychology. *Couns Psychol Q.* (2015) 28(3):324-344. https://doi.org/10.1080/09515070.2015.1053433
- McCrickard, M.P., & Butler, L.T. Cybercounseling: A new modality for counselor training and practice. *Int J Adv Couns.* (2005) 27(1):101-110. https://doi.org/10.1007/s10447-005-2255-x
- McInroy, L.B. Teaching technology competencies: A social work practice with technology course. *J Soc Work Educ.* (2021) 57(3):545-556. https://doi.org/10.1080/10437797.2019.1491272
- Menzano, S., et al. Practical, ethical, and legal considerations regarding videocounseling in college and university counseling centers: A response to Quarto's 'influencing college students' perceptions of videocounseling. *J Coll Stud Psych.* (2011) 25:326-333. https://doi.org/10.1080/87568225.2011.605695
- Merrill, C.A., et al. CtiBS and clinical social work: Telebehavioral health competencies for LCSWs in the age of COVID-19. *Clin Soc Work J.* (2022) 50:115-123. https://doi.org/10.1007/s10615-021-00827-7
- Miller, T.W., et al. Telepsychiatry: Critical dimensions for forensic services. *J AM Acad Psychiatry Law.* (2005) 33:539-546.
- Miller, T.W., et al. Teleconferencing model for forensic consultation, court testimony, and continuing education. *Behav Sci Law.* (2008) 26:301-313. https://doi.org/10.1002/bsl.809
- Misra, U.K., et al. Telemedicine in neurology: Underutilized potential. *Neurol India.* (2005) 53(1):27-31. https://doi.org/10.4103/0028-3886.15047
- Murphy, J.M., & Pomerantz, A.M. Informed consent: An adaptable question format for telepsychology. *Prof Psychol Res Pr.* (2016) 47(5):330-339. https://doi.org/10.1037/pro0000098
- Nelson, E-L., & Velasquez, S.E. Implementing psychological services over televideo. *Prof Psychol Res Pr.* (2011) 43(6):535-542. https://doi.org/10.1037/a0026178
- Newby, J., et al. Integrating internet CBT into clinical practice: A practical guide for clinicians. *Clin Psychol.* (2021) 25(2):164-178. https://doi.org/10.1080/13284201.2020.1843968
- Noronha, C., et al. Telehealth competencies in medical education: New frontiers in faculty development and learner assessments. *J Gen Intern Med.* (2022) 37:3168-3173. https://doi.org/10.1007/s11606-022-07564-8
- Panos, P.T., et al. Ethical issues concerning the use of videoconferencing to supervise international social work field practicum students. *J Soc Work Educ.* (2002);38(3):421-437. https://doi.org/10.1080/10437797.2002.10779108
- Parish, M.B., et al. Asynchronous telepsychiatry interviewer training recommendations: A model for interdisciplinary, integrated behavioral health care. *Telemed J e-Health.* (2021) 27(9):982-988. https://doi.org/10.1089/tmj.2020.0076
- Patel, Z.S., Tarlow, N., & Tawfik, S.H. Assessment supervision during COVID-19 and beyond: Trainee perspectives on the supervision of teleassessment. *Train Educ Prof Psychol.* (2021) 15(4):276-283. https://doi.org/10.1037/tep0000342
- Perle, J.G. Introduction to telehealth for clinical psychologists: A novel course designed to improve general knowledge and hands-on expertise with technology-based modalities. *J Technol Behav Sci.* (2020) 5:383-394. https://doi.org/10.1007/s41347-020-00147-6

- Perle, J.G., et al. Educating for the future: A preliminary investigation of doctoral-level clinical psychology training program's implementation of telehealth education. *J Technol Behav Sci.* (2022) 7:351-357. https://doi.org/10.1007/s41347-022-00255-5
- Perle, J.G., et al. Fostering telecompetence: A descriptive evaluation of clinical psychology predoctoral internship and postdoctoral fellowship implementation of telehealth education. *J Rural Health.* (2023) 39:444-451. https://doi.org/10.1111/jrh.12709
- Phillips, L.A., Logan, J.N., & Mather, D.B. COVID-19 and beyond: Telesupervision training within the supervision competency. *Train Educ Prof Psychol.* (2021) 15(4):284-289. https://doi.org/10.1037/tep0000362
- Prabhakar, E. e-Therapy: Ethical considerations of a changing healthcare communication environment. *Pastoral Psychol.* (2013) 62:211-218. https://doi.org/10.1007/s11089-012-0434-3
- Qureshi, A.Z., et al. Telerehabilitation guidelines in Saudi Arabia. *Telemed J e-health.* (2021) 27(10):1087-1098. https://doi.org/10.1089/tmj.2020.0355
- Rabe, M. Telehealth in South Africa: A guide for healthcare practitioners in primary care. *S Afr Fam Pract.* (2022) 64(1):a5533. https://doi.org/10.4102/safp.v64i1.5533
- Reamer, F.G. Social work in a digital age: Ethical and risk management challenges. *Soc Work.* (2013) 58(2):163-172. https://doi.org/10.1093/sw/swt003
- Rees, C.S, & Haythornthwaite, S. Telepsychology and videoconferencing: Issues, opportunities and guidelines for psychologists. *Aust Psychol.* (2004) 39(3):212-219. https://doi.org/10.1080/00050060412331295108
- Rezai-Rad, M., Vaezi, R., & Nattagh, F. e-Health readiness assessment framework in Iran. *Iranian J Publ Health.* (2012) 41(10):43-51.
- Roth, L.T., et al. Can you hear me now? A toolkit for telemedicine training. *Clin Teach.* (2021) 18:348-353. https://doi.org/10.1111/tct.13390
- Rutledge, C.M., et al. Telehealth and e-health in nurse practitioner training: Current perspectives. *Adv Med Educ Pract.* (2017) 8:399-409.
- Rutledge, C.M., et al. Educating advanced practice nurses in using social media in rural health care. *Int J Nurs Educ Scholarsh.* (2011) 8(1):Article 25. https://doi.org/10.2202/1548-923X.2241
- Sabin, J.E., & Skimming, K. A framework of ethics for telepsychiatry practice. *Int Rev Psychiatry.* (2015) 27(6):490-495. https://doi.org/10.3109/09540261.2015.1094034
- Saeed, S.A., et al. Training residents in the use of telepsychiatry: Review of the literature and a proposed elective. *Psychiatr Q.* (2017) 88(2):271-283. https://doi.org/10.1007/s11126-016-9470-y
- Schwartz, T.J., & Lonborg, S.D. Security management in telepsychology. *Prof Psychol Res Pr.* (2011) 42(6):419-425. https://doi.org/10.1037/a0026102
- Shandley, K., et al. Training postgraduate psychology students to deliver psychological services online. *Aust Psychol.* (2011) 46:120-125. https://doi.org/10.1111/j.1742-9544.2011.00034.x
- Sherbersky, H., Ziminski, J., & Pote, H. The journey towards digital systemic competence: Thoughts on training, supervision and competence evaluation. *J Fam Ther.* (2021) 43:351-371. https://doi.org/10.1111/1467-6427.12328

- Simpson, S.G., et al. Tele-web psychology in rural South Australia: The logistics of setting up a remote university clinic staffed by clinical psychologists in training. *Aust Psychol.* (2014) 49:193-199. https://doi.org/10.1111/ap.12049
- Smith, T., Norton, A.M., & Marroquin, L. (2023). Virtual family play therapy: A clinician's guide to using directed family play therapy in telemental health. *Contemp Fam Ther.* (2023) 45:106-116. https://doi.org/10.1007/s10591-021-09612- 7
- Spelten, E.R., et al. Best practice in the implementation of telehealth-based supportive cancer care: Using research evidence and discipline-based guidance. *Patient Educ Couns.* (2021) 104:2682-2699. https://doi.org/10.1016/j.pec.2021.04.006
- Stoll, J., Muller, J.A., & Trachsel, M. Ethical issues in online psychotherapy: A narrative review. *Front Psychiatry.* (2020) 10(993):498439. https://doi.org/10.3389/fpsyt.2019.00993
- Strowd, R.E., et al. Practical guidance for telemedicine use in neuro-oncology. *Neurooncol Pract.* (2022) 9(2):91-104. https://doi.org/10.1093/nop/npac002
- Sunderji, N., Crawford, A., & Jovanovic, M. Telepsychiatry in graduate medical education: A narrative review. *Acad Psychiatry.* (2015) 39:55-62. https://doi.org/10.1007/s40596-014-0176-x
- Taylor, J., & Fuller, B. The expanding role of telehealth in nursing: Considerations for nursing education. *Int J Nurs Educ Scholarsh.* (2021) 18(1):20210037. https://doi.org/10.1515/ijnes-2021-0037
- Townsend, B.A., Scott, R.E., & Mars, M. The development of ethical guidelines for telemedicine in South Africa. *S Afr J Bioethics Law.* (2019) 12(1):19-26. https://doi.org/10.7196/SAJBL.2019.v12i1.662
- Webb, C., & Orwig, J. Expanding our reach: Telehealth and licensure implications for psychologists. *J Clin Psychol Med Settings.* (2015) 22:243-250. https://doi.org/10.1007/s10880-015-9440-9
- Weisenmuller, C.M., & Luzier, J.L. Technology is a core competency in professional psychology. *Train Educ Prof Psychol.* (2023) 17(3):241-247. https://doi.org/10.1037/tep0000423
- Yellowlees, P.M., Holloway, K.M., & Parish, M.B. Therapy in virtual environments – Clinical and ethical issues. *Telemed J e-Health.* (2012) 18(7):558-564. https://doi.org/10.1089/tmj.2011.0195
- Zha, A.M., et al Training in neurology: Adoption of resident teleneurology training in the wake of COVID-19. *Neurol.* (2020) 95(9):404-407. https://doi.org/10.1212/WNL.0000000000010029
- Ziade, N., et al. Telehealth in rheumatology: The 2021 Arab League of Rheumatology best practice guidelines. *Rheumatol Int.* (2022) 42:379-390. https://doi.org/10.1007/s00296-021-05078-w
- Zickuhr, L., et al. Addressing competency in rheumatology telehealth care delivery. *Arthritis Care Res.* (2023) 75(6):1213-1219. https://doi.org/10.1002/acr.25023
